# Haploinsufficiency of Hedgehog interacting protein causes increased emphysema induced by cigarette smoke through network rewiring

**DOI:** 10.1186/s13073-015-0137-3

**Published:** 2015-02-14

**Authors:** Taotao Lao, Kimberly Glass, Weiliang Qiu, Francesca Polverino, Kushagra Gupta, Jarrett Morrow, John Dominic Mancini, Linh Vuong, Mark A Perrella, Craig P Hersh, Caroline A Owen, John Quackenbush, Guo-Cheng Yuan, Edwin K Silverman, Xiaobo Zhou

**Affiliations:** Channing Division of Network Medicine, Department of Medicine, Brigham and Women’s Hospital and Harvard Medical School, Boston, MA USA; Department of Biostatistics and Computational Biology, Dana-Farber Cancer Institute, Boston, MA USA; Department of Biostatistics, Harvard School of Public Health, Boston, MA USA; Division of Pulmonary and Critical Care Medicine, Department of Medicine, Brigham and Women’s Hospital and Harvard Medical School, Boston, MA USA; The Lovelace Respiratory Research Institute, Albuquerque, NM USA; Pulmonary Department, University of Parma, Parma, Italy; Pediatric Newborn Medicine, Brigham and Women’s Hospital and Harvard Medical School, Boston, MA USA

## Abstract

**Background:**

The *HHIP* gene, encoding Hedgehog interacting protein, has been implicated in chronic obstructive pulmonary disease (COPD) by genome-wide association studies (GWAS), and our subsequent studies identified a functional upstream genetic variant that decreased *HHIP* transcription. However, little is known about how *HHIP* contributes to COPD pathogenesis.

**Methods:**

We exposed *Hhip* haploinsufficient mice (*Hhip*^*+/-*^) to cigarette smoke (CS) for 6 months to model the biological consequences caused by CS in human COPD risk-allele carriers at the *HHIP* locus. Gene expression profiling in murine lungs was performed followed by an integrative network inference analysis, PANDA (Passing Attributes between Networks for Data Assimilation) analysis.

**Results:**

We detected more severe airspace enlargement in *Hhip*^*+/-*^ mice vs. wild-type littermates (*Hhip*^*+/+*^) exposed to CS. Gene expression profiling in murine lungs suggested enhanced lymphocyte activation pathways in CS-exposed *Hhip*^*+/-*^ vs. *Hhip*^*+/+*^ mice, which was supported by increased numbers of lymphoid aggregates and enhanced activation of CD8+ T cells after CS-exposure in the lungs of *Hhip*^*+/-*^*mice* compared to *Hhip*^*+/+*^ mice. Mechanistically, results from PANDA network analysis suggested a rewired and dampened Klf4 signaling network in *Hhip*^*+/-*^ mice after CS exposure.

**Conclusions:**

In summary, *HHIP* haploinsufficiency exaggerated CS-induced airspace enlargement, which models CS-induced emphysema in human smokers carrying COPD risk alleles at the *HHIP* locus. Network modeling suggested rewired lymphocyte activation signaling circuits in the *HHIP* haploinsufficiency state.

**Electronic supplementary material:**

The online version of this article (doi:10.1186/s13073-015-0137-3) contains supplementary material, which is available to authorized users.

## Background

Chronic obstructive pulmonary disease (COPD), the third leading cause of death in the US [1], is a complex disease strongly influenced by cigarette smoke (CS) and genetic predisposition [2,3]. COPD is characterized by emphysematous destruction of the alveoli and thickening of the small airway walls in response to chronic exposure to cigarette smoke (CS). While the cause of emphysematous destruction of the alveoli is likely due to a combination of the cytotoxic and pro-inflammatory activities of CS, the pathogenic mechanisms underlying the disease remain inadequately defined [4].

Recently, progress in genome-wide association studies (GWAS) has provided compelling evidence for several COPD susceptibility loci, including an intergenic region on chromosome 4q31 [5,6]. Subsequent work by our group confirmed hedgehog interacting protein (*HHIP*) as the causative gene for this locus and identified a potential functional variant [7]. HHIP is a negative regulator of the Hedgehog pathway that is important for morphogenesis of the lungs and other organs [8-10]. HHIP competitively binds to all three ligands of the Hedgehog pathway: Sonic Hedgehog (Shh), Indian Hedgehog (Ihh), and Desert Hedgehog (Dhh). Recent reports have suggested potential roles for the Hedgehog pathway in bleomycin-induced pulmonary fibrosis [11] and CS-induced lung cancer [12]. However, the mechanisms by which *HHIP* influences COPD susceptibility remain to be determined. We have now extended our previous work to an *in vivo* murine model by demonstrating that *Hhip* haploinsufficiency exaggerated CS-induced airspace enlargement and rewired lymphocyte activation signaling pathways under CS exposure.

## Methods

### Mice and cigarette smoke exposure in mice

*Hhip*^*+/-*^ mice in a mixed genetic background (Jackson Laboratory) were backcrossed for 12 generations to C57BL/6J wild-type mice that were purchased from Jackson Laboratory. All mice were housed in the animal facility at Harvard Medical School with a 12 h light/12 h dark cycle. This study was performed in strict accordance with the recommendations in the Guide for the Care and Use of Laboratory Animals of the National Institutes of Health. The protocol was approved by the Harvard Medical Area (HMA) Standing Committee on Animals (Protocol Number: 04833). Every effort was made to minimize suffering.

Female *Hhip*^*+/-*^ and littermate mice (approximately 10 weeks old) were exposed to mixed main-stream and side-stream CS from 3R4F Kentucky Research cigarettes for 5 days/week in Teague TE 10z chambers (Total Suspended Particulates approximately 100 to 200 mg/m^3^ and CO levels approximately 6 ppm). As a control, mice were exposed to filtered air for the same duration. At the end of the 6-month exposure period, both respiratory mechanics and airspace enlargement were measured in mice exposed to CS or air. All mice were euthanized by CO_2_ narcosis and cervical dislocation before removing lungs for other studies.

### Measurement of lung mechanics

To measure respiratory mechanics, mice were anesthetized with a cocktail of 100 mg/kg body weight ketamine, 10 mg/kg xylazine, and 3 mg/kg acepromazine. A tracheostomy was performed, and an 18-gauge cannula was inserted and secured in the trachea using sutures. Mice were then connected via the cannula to a digitally controlled mechanical ventilator (Flexi Vent device; Scireq Inc., Montreal, QC, Canada). Ventilator settings were *f* = 150/min, FiO_2_ = 0.21, tidal volume = 10 mL/kg body weight, and positive end-expiratory pressure (PEEP) = 3 cm H_2_O. Murine lungs were inflated to total lung capacity (TLC; 25 cm H_2_O) three times for a volume history. Quasi-lung compliance and tissue elastance at PEEP of 3 cm H_2_O were then measured.

### Morphometric analysis of airspace size in mice

After completion of the respiratory mechanics measurements, mice were euthanized; lungs were inflated with PBS to a constant pressure of 25 cm H_2_O and then fixed with 10% formaldehyde for 48 h. The mean alveolar chord length was measured after slides were stained with Gill’s solution as described previously [13]. At least 15 images per mouse lung were randomly captured for analysis using methods described previously [14]. Images were processed and analyzed for mean alveolar chord length using Scion image software.

### Gene expression profiling

Six mice from each of four groups with different genotypes (*Hhip*^*+/+*^ or *Hhip*^*+/-*^) and treatments (air or CS) were randomly chosen for gene expression profiling. RNA was extracted from murine lung tissues using Allprep kit (Qiagen, Valencia, CA, USA); 750 ng of total RNA for each sample was hybridized onto a Sentrix MouseRef-8 v2.0 Expression BeadChip Array (Illumina, San Diego, CA, USA), based on the manufacturer’s protocol. The BeadChip Array was scanned using an Illumina BeadArray Reader and quality control analysis was performed using Illumina GenomeStudio v3.1.3 software, Gene Expression Module version 1.1.1, and the statistical package R v2.15.2. Each of the samples and all probes passed quality control, and the data were background corrected, log2 transformed, and quantile normalized. Only probes with gene symbol and chromosome number annotations were retained, providing 22,121 of the 25,697 probes for analysis. A linear regression model was fit to the data for each probe to detect differential expression between genotypes (*Hhip*^*+/+*^/*Hhip*^*+/-*^) and treatments (air/CS), and having the processing information available for each array. The chip barcode was included as a covariate in the model to correct for batch effects. Clustering and MDS plots provided evidence of acceptable batch correction. In order to provide insight into probes demonstrating differential expression in *Hhip*^*+/+*^ and *Hhip*^+/-^ mice in response to air/CS treatment, the interaction term genotype*treatment was included in the linear model. The genotype-by-treatment interaction was evaluated using the linear model expression ~ treatment + genotype + treatment*genotype + batch. Lastly, to stabilize the standard error of the estimated regression coefficient, we applied an empirical Bayes shrinkage method to obtain a moderated t-test statistic and its *P* value (R package limma v3.14.3). To adjust for multiple testing and to control false discovery rate (FDR), we corrected the *P* values using the Benjamin and Hochberg (1995) FDR correction method. Genes with *P* adjusted <0.05 were defined as significantly different gene expression. The network-based functional prediction was performed on the GeneMANIA webserver [15,16] using the default list of networks and weighting.

The microarray data have been deposited in NCBI’s Gene Expression Omnibus [17] and are accessible through GEO Series accession number GSE65124.

### Detection of gene expression by real-time RT-PCR

Real-time PCR with gene-specific TaqMan primers/probes was performed as described previously [18]. Relative gene expression was calculated based on the standard 2^-ΔΔCT^ method, using *GAPDH* (glyceraldehyde 3-phosphate dehydrogenase) as a reference gene. Gene expression levels were analyzed by both two-way ANOVA analysis and student two-sample unpaired *t* test.

### Quantification of lymphoid aggregates in murine lungs exposed chronically to cigarette smoke

The number of lymphoid aggregates was determined by counting total number of lymphoid aggregates around airways with 200 to 1,000 μm diameter in each murine lung section that was stained with hematoxylin and eosin. The correlation between the number of lymphoid aggregates per airway and airspace size represented by mean airspace chord length (MCL) was determined by Pearson’s correlation coefficient using commercial statistical software STATA (version 12.1). A scatter plot analysis of MCL vs. lymphoid aggregates per airway was performed in the four groups of mice.

### Immunohistochemistry staining of T cells and B cells in lung sections

We performed triple immunofluorescence staining of lung sections from Hhip^+/-^ mice exposed to CS for 6 months. Briefly, slides were deparaffinized, and antigen retrieval was achieved by heating slides in 10 mM sodium citrate and 2 mM citric acid buffer (pH 6.0). Lung sections were incubated for 1 h at 37°C with goat anti-CD4 IgG (Abcam, Cambridge, MA, USA; diluted 1:50), then for 1 h at 37°C with rat anti-CD8 IgG (Novus Biologicals, Littleton, CO, USA; diluted 1:100), and then 1 h at 37°C with rabbit anti-CD20 IgG (Abcam, Cambridge, MA, USA; diluted 1:100) followed by incubation with second antibodies including Alexa 647-conjugated rabbit anti-goat IgG (diluted 1:100), Alexa 488-conjugated goat anti-rat F(ab')_2_ (diluted 1:100), and Alexa 546-conjugated goat anti-rabbit IgG (diluted 1:100). Images of the stained lung sections were analyzed with a confocal microscope (Leica Microsystems, Buffalo Grove, IL, USA).

### Quantification of CD8+ T cells in lung parenchyma

The number of CD8+ T cells was measured in lung sections from *Hhip*^*+/+*^ and *Hhip*^*+/-*^ mice exposed to air (n = 4 mice/group) or CS (n = 5 *Hhip*^*+/+*^ and n = 9 *Hhip*^*+/-*^ mice) for 6 months. After antigen retrieval in 10 mM sodium citrate and 2 mM citric acid buffer (pH 6.0), lung sections were incubated with a rat IgG to murine CD8 (Novus Biologicals, Littleton, CO, USA; diluted 1:100) or non-immune murine IgG as control for 1 h at 37°C. After secondary antibody incubation and counterstaining with DAPI, the number of CD8 positive cells was then normalized by unit of alveolar area that was quantified using MetaMorph software (Molecular Devices, Wetzlar, Germany) and compared between groups using two-way ANOVA analysis and two-sided unpaired t-test.

### Measurement of cytokines by enzyme-linked immunosorbent assay (ELISA)

Interleukin 2 (IL-2) and Interferon gamma (IFN-γ) levels in murine lungs were measured using mouse Quantikine ELISA Kit and mouse IFN-γ Duoset (R&D, Minneapolis, MN, USA) following the manufacturer’s protocol and then normalized to total lung protein amount measured using Bradford Assay (Biorad, Hercules, CA, USA).

### Leukocyte counting in bronchoalveolar lavage samples after acute smoke exposure

*Hhip*^*+/+*^ and *Hhip*^*+/-*^ mice were exposed to CS or air for 2 months and then bronchoalveolar lavage was performed using 3 x 1 mL aliquots of PBS. Total leukocyte counts were performed using a hemocytometer.

### Flow cytometric analysis to quantify activation of T lymphocytes

Immediately after CS exposure and lung removal, lung tissues were digested with collagenase and DNase (Sigma, St. Louis, MO, USA) to prepare single-cell suspensions by passing the dissociated tissue through a 70-μm cell strainer (BD Falcon). The suspensions of cells were stained in PBS containing 2% FBS with the following antibodies: FITC-conjugated anti-CD4, PE-Cy7-conjugated anti-CD3, APC-Cy7-conjugated anti-CD45, PE-conjugated anti-CD69, and APC-conjugated anti-CD8 antibodies (BD Bioscience). The cells were gated on FSC (forward scatter) and SSC (side scatter). The cells were then gated on SSC versus CD45 followed by SSC versus CD3 in order to gate on all T cells. The cells were further gated to obtain the percentage of CD4+ T and CD8+ T cells. Stained cells were examined on a FACS Canto flow cytometer (BD Bioscience, San Jose, CA, USA) and analyzed using FlowJo software (TreeStar, Ashland, OR, USA). The percentages of activated CD4+ T cells and activated CD8+ T cells were determined based on double positive cells (CD69+ CD4+ or CD69+ CD8+ cells) among total CD4+ or CD8+ T cells.

### Network building

We used the ‘PANDA’ method [19] to construct four genome-wide regulatory network models based on *Hhip* genotype (*Hhip*^*+/-*^ or *Hhip*^*+/+*^) and treatment (CS or Air). To begin, we collapsed expression values for probe sets that map to the same gene symbol by selecting, for each experiment, the signal from the probe set with the most significant detection *P* value, resulting in expression information for 15,124 unique genes. We then obtained the position-weight-matrix for 374 unique vertebrate TFs with known binding site motifs, 130 recorded in the Jaspar database [20], and 290 from the uniprobe database [21] (46 TFs have motifs in both databases). Defining a ‘true hit’ as a log-odds ratio greater than 7.5, we used HOMER [22] to map each of these motifs to putative regulatory regions, defined as (-750, +250) base-pairs around the transcription start site, for 15,124 genes with expression data in the samples. Because transcriptional regulation involves assembly of protein complexes and allows for combinatorial regulatory processes, we also collected information regarding physical protein interaction data between transcription factors estimated from mouse-2-hybrid analysis [23]. Finally, we used PANDA [19] to integrate information from transcription factor binding motifs, protein-protein interaction data, and gene expression for each experimental condition, and constructed directed networks for each experimental condition. To reduce potential background noise due to microarray sample size (6 mice/group), we applied a jack-knife [24] method to repeatedly sample four arrays out of six from each experimental condition (*Hhip*^*+/+*^*-*Air or CS and *Hhip*^*+/-*^ Air or CS), building 15 networks per group.

After reconstructing the networks, we specifically investigated the differential targeting of HHIP in the predicted networks. To begin, we identified 32 transcription factors that have a putative target in the promoter region of *HHIP*. We then identified the 32 ‘edges’ that extend between these transcription factors and *HHIP* in our reconstructed network models. To evaluate whether any of these 32 edges are significantly different between the sets of networks associated with each of the experimental conditions, we performed an unpaired two-sample t-test, comparing the distribution of edge weights in each pair of conditions.

We next identified subnetworks of edges that are most distinct between each pair of our reconstructed network models. For each edge connecting a transcription factor to its target gene, PANDA assigns a Z-score weight reflecting the confidence level of the inferred regulatory relationship. We contrasted both the average and the distribution (using an unpaired two sample t-test) of this weight value between sets of networks (those reconstructed for each experimental condition using the jack-knife procedure, see above). Using this information, we identified subnetworks of edges that differ between each pair of networks by selecting high confidence edges, defined as those with an absolute difference in average weight greater than 3 and a *P* value from the t-test of less than 1×10^-3^. We used DAVID [25] to test for pathway enrichment in genes targeted in each of these identified subnetworks. The *P* values for KEGG Pathways enriched at a Benjamini-Hochberg significance of less than 0.01 in at least one of these subnetworks were illustrated in a heat map. At this confidence level the selected pathways all had an overlap of greater than 10 genes with their respective differentially targeted gene sets.

We also characterized the differential targeting patterns of transcription factors in these pairs of subnetworks by calculating, for each transcription factor, a fold-enrichment for its number of outgoing edges in one subnetwork compared to the other, as well as an associated *P* value. To calculate the *P* value we performed Fisher’s exact test and determined the significance of the overlap in the transcription factor’s outgoing edges with the edges in its associated ‘enriched’ subnetwork, using edges in either subnetwork as a background. A list of core TFs for each of the subnetworks was then generated by filtering TFs with total edges > =3 and absolute log2 fold change greater than 1 or -log10 *P* value greater than 3 in either subnetwork. Finally, we took the union of these core TFs and determined their average expression across samples in each of the four experimental groups. We also used an unpaired t-test to evaluate their pairwise differential expression between the groups. Note that several of these core TFs identified through the network analysis (which utilizes motif information) did not have probes on the array, including ELK1, SOX11, and ZFP740, and these are excluded from expression analysis.

### Statistical analysis methods

For MCL analysis, we applied a linear mixed effects model for mice that were exposed to CS, in which the probit-transformed lung damage (quantified alveolar chord length for each image from each mouse) is the outcome variable, the intercept is the random effect to account for the dependence among the longitudinal measurements of the lung damage measurement, and mice type is the fixed effect (wild-type mice as the reference group). We used two-way ANOVA analysis first to determine whether there are overall differences among all four groups of mice followed by unpaired t tests to determine whether there are significant differences between each pair of groups. For gene expression of Gstp1, Cstw, Fyb, Lat2, and Klf4, we first evaluated if there is an interaction effect between genotype and treatment on gene expression using two-way ANOVA analysis. We then compared the two conditions of a factor (genotype or CS treatment) for a given level of the other factor (CS treatment or genotype) using un-paired two sample two-sided t tests. However, for statistical analysis on CD8+ T cells quantification, to handle the non-normality due to outliers we applied Kruskal-Wallis test, which is a non-parametric test without assumption of normality, and detected significant differences among groups (*P* <0.001). We then applied Wilcoxon rank sum test for pair-wise comparisons. All other data were also analyzed by both two-way ANOVA analysis and student two-sample unpaired *t* tests.

## Results and Discussion

### *Hhip*^*+/-*^ mice demonstrated more severe airspace enlargement induced by cigarette smoke compared with wild-type littermate control mice

**Figure 1:**
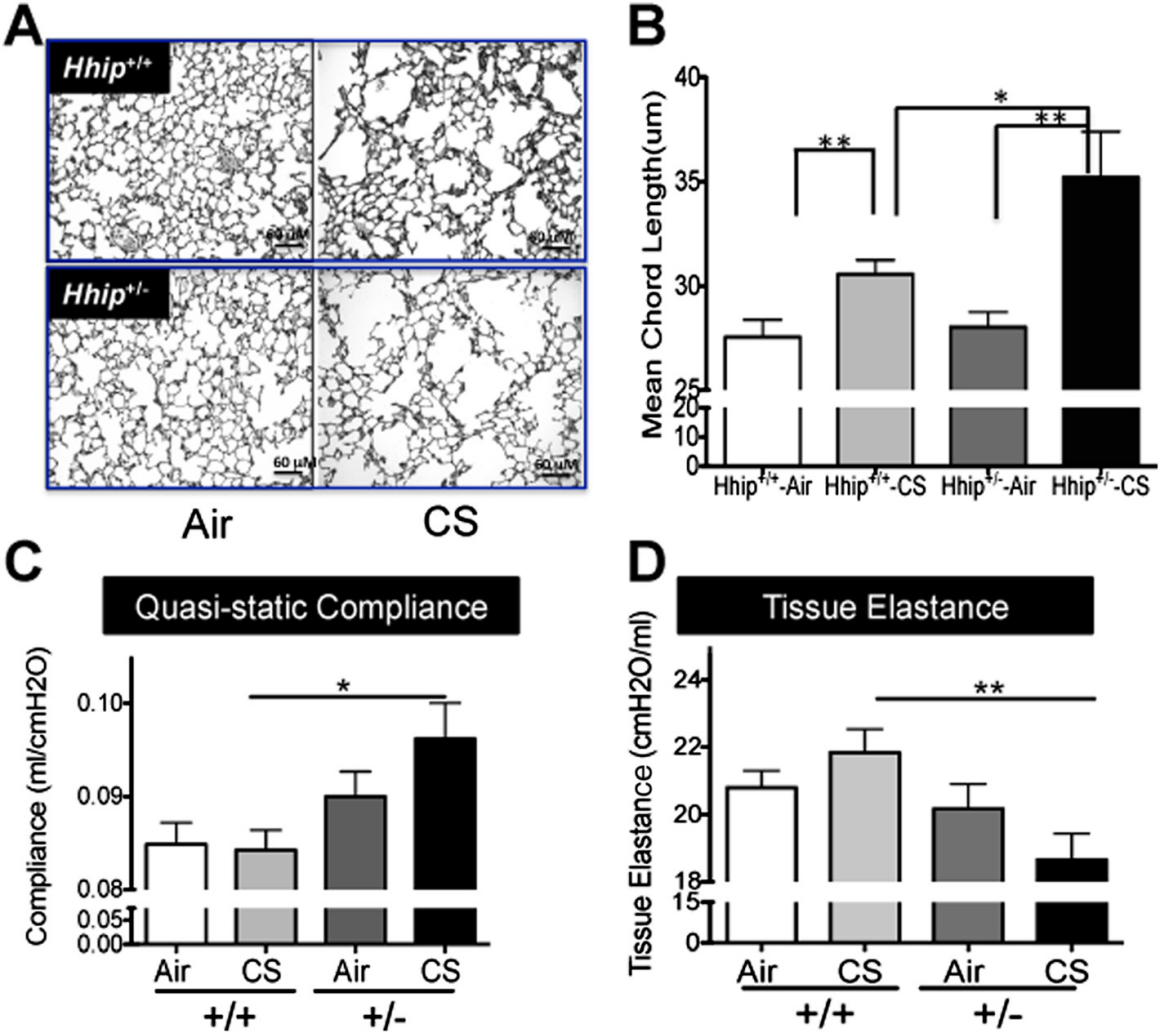
***Hhip*** ^***+/-***^ **mice demonstrated more severe airspace enlargement induced by cigarette smoke compared with wild-type littermate control mice.** Airspace enlargement of Hhip^+/-^ vs. wild-type littermate control mice (Hhip^+/+^) exposed to cigarette smoke (CS) or air for 6 months as demonstrated by Gill staining **(A)** and measured by mean alveolar chord length, MCL **(B)**. Mean ± SEM is shown in panel B from 11 to 19 mice per group. Lung mechanics measurements revealed increased lung compliance **(C)** and decreased tissue elastance **(D)** in Hhip^+/-^ mice exposed to CS compared to CS-exposed Hhip^+/+^ mice. *P <0.05 and **P <0.01. Mean ± SEM are shown in each group.

Previously, we detected approximately 32% reduced gene expression of *HHIP* in human COPD lung tissues compared with ex-smoker controls with normal lung function [7]. Moreover, we found that genetic variants in the chromosome 4q31 COPD GWAS region [7] moderately regulate expression of *HHIP. Hhip*^-/-^ mice die shortly after birth due to respiratory failure secondary to defects in lung branching morphogenesis [10] and, thus, cannot be used to assess the effects of *Hhip* on COPD pathogenesis. In contrast, *Hhip*^+/-^ mice, with only modestly reduced expression of *Hhip* (approximately 33%) in murine lungs (Additional file 1: Figure S1), have normal survival and normal lung development [10]. Thus, *Hhip*^+/-^ mice may be more suitable than *Hhip*^-/-^ mice for recapitulating the biological consequences caused by human functional genetic variants *in vivo. Hhip*^+/-^ mice in a C57BL/6 J background were exposed to CS or room air for 6 months with wild-type littermate control mice (*Hhip*^*+/+*^). Mean alveolar chord length (MCL) was measured in all groups as described previously [13]. *Hhip*^+/-^-CS mice had significantly increased airspace size compared with CS-exposed *Hhip*^*+/+*^mice (Figure 1A, B, *P* <0.05), while air-exposed *Hhip*^*+/-*^ and *Hhip*^*+/+*^ mice had similar distal airspace size assessed using either two-way ANOVA or unpaired t test.

For lung mechanics, CS-exposed *Hhip*^*+/-*^ mice had increased quasi-static lung compliance (Figure 1C) and correspondingly decreased tissue elastance (Figure 1D) compared to CS-exposed *Hhip*^*+/+*^ mice, indicating that *Hhip*^*+/-*^ mice exposed to CS have more compliant lungs and a greater loss of elastic recoil than *Hhip*^*+/+*^ mice exposed to CS.

### Assessments of the Hedgehog pathway in murine lungs

Given that *HHIP* is known to inhibit the Hedgehog pathway, we assessed the expression of major components in the Hedgehog pathway in the chronic CS exposure murine model. Interestingly, Gli1 and Patch1 showed increased expression in air-exposed *Hhip*^*+/-*^ mice vs. air-exposed *Hhip*^*+/+*^ mice while Gli1 and Gli2 showed increased expression in CS-exposed *Hhip*^*+/-*^ mice vs. CS-exposed *Hhip*^*+/+*^ mice. No significant differences in Gli3 were observed among these four groups of mice (Additional file 1: Figure S2). Hence, *Hhip*^*+/-*^ mice may have relatively higher activity of the Hedgehog pathway at baseline independent of CS treatment.

### Gene expression profiling in murine lungs after cigarette smoke exposure

In order to identify molecular targets that contributed to increased airspace size in *Hhip*^*+/-*^ mice exposed to CS, we performed gene expression profiling in lung tissues from randomly chosen *Hhip*^*+/-*^ and *Hhip*^*+/+*^ mice exposed to either air or CS for 6 months (n = 6 mice/group). Data were preprocessed using the LIMMA package in R/Bioconductor, as we have done previously [17].

**Figure 2:**
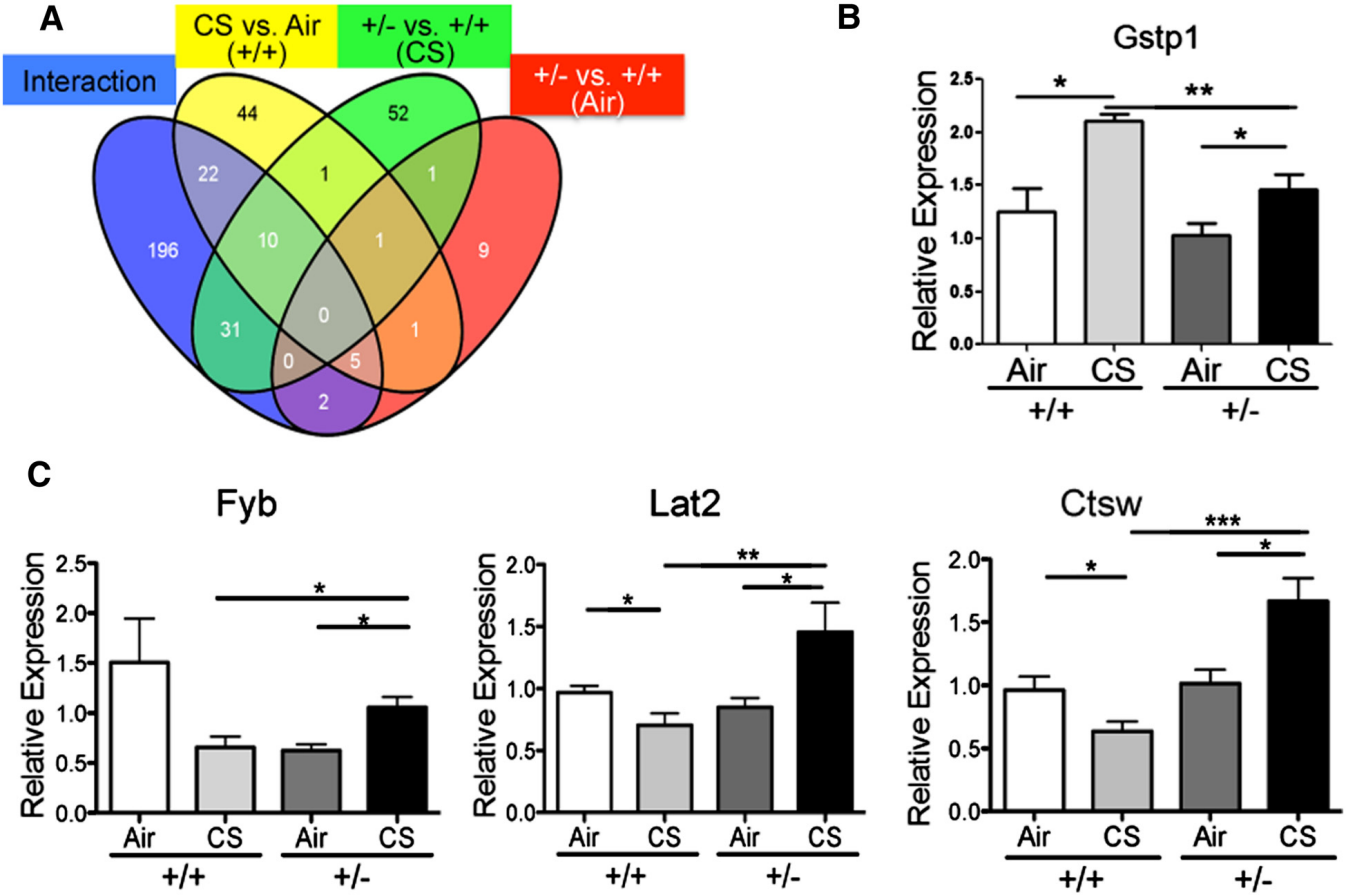
**Gene expression profiling in lungs from** ***Hhip*** ^***+/-***^ **and** ***Hhip*** ^***+/+***^ **control mice exposed to either room air (Air) or cigarette smoke (CS) for 6 months. (A)** Venn diagram shows comparisons of differentially expressed genes in *Hhip* ^*+/+*^-CS vs. *Hhip* ^*+/+*^-Air (yellow oval), *Hhip* ^*+/+*^-CS vs. *Hhip* ^*+/-*^-CS (green oval), *Hhip* ^*+/-*^-Air vs. *Hhip* ^*+/+*^-Air (red oval) and genes enriched based on interaction with smoke treatment (blue oval). **(B)** Real-time PCR showed differential gene expression of Gstp1 and also of a subset of genes **(C)** revealed by gene-by-treatment interaction analysis. Relative gene expression levels were expressed as fold changes after normalized to Gapdh (glyceraldehyde 3-phosphate dehydrogenase) as the reference gene. Mean ± SEM are shown; n = 6 mice/group. Unpaired t test. *P <0.05; **P <0.01. ***P <0.001.

In addition to identification of differentially expressed genes in each group by pairwise comparisons (Additional file 2: Table S1A and Additional file 3: Table S1B), we also set out to identify genes that responded to CS differentially in *Hhip*^*+/-*^ and *Hhip*^*+/+*^ mice using linear regression models with an interaction term between genotype and CS treatment in all groups. There were 266 genes that showed significant genotype-by-CS treatment interaction (*P* adj <0.05, indicated by the blue oval in Figure 2A, Additional file 4: Table S2A). Furthermore, these 266 genes are enriched for B and T cell activation and positive regulation of immune response as revealed by GeneMANIA analysis [15,16] (Additional file 2: Table S2B), suggesting an unexpected role of HHIP in lymphocyte activation. Furthermore, 41 out of these 266 genes (Additional file 2: Table S3A) also showed differential gene expression in *Hhip*^*+/+*^-CS vs. *Hhip*^*+/-*^-CS group comparisons (overlap between blue and green oval in Figure 2A) that are also enriched in lymphocyte activation by pathway analysis (Additional file 2: Table S3B). To exclude possible bias resulting from different numbers of genes in each comparison, we also used probe sets to perform these comparisons. A similar number of genes were identified when probe sets instead of genes were used (Additional file 1: Figure S3).

We then validated expression changes of 16 genes that demonstrated significant gene-by-CS treatment interactions and also were related to lymphocyte activation pathways by real-time RT-PCR (Additional file 2: Table S4). One gene, Gstp1 (Glutathione S-Transferase P1), which showed differential expression between both *Hhip*^*+/-*^*-*CS vs. *Hhip*^*+/+*^*-*CS (green in Figure 2A) and *Hhip*^*+/+*^*-*CS vs. *Hhip*^*+/+*^*-*air (yellow in Figure 2A), demonstrated diminished induction by CS in *Hhip*^*+/-*^ vs. *Hhip*^*+/+*^ mice (Figure 2B). Moreover, several genes that were identified as having significant genotype-by-treatment interactions and were related to lymphocyte activation showed increased expression in the lungs of *Hhip*^*+/-*^*-*CS compared to *Hhip*^*+/+*^-CS mice (Figure 2C) and significant genotype-by-treatment interaction as suggested by two-way ANOVA analysis (Figure 2C, *P* <0.01). For example, Fyb (Fyn binding protein), also called ADAP (adhesion and degranulation-promoting adapter protein), promotes T cell conjugation to antigen-laden antigen presenting cells (APC) and enhances T cell survival [26]. Linker for activation of T cells family member 2 (Lat2) showed significantly increased expression in the lungs of *Hhip*^*+/-*^*-*CS compared to *Hhip*^*+/+*^-CS mice, in contrast to slightly reduced expression in *Hhip*^*+/+*^ mice after CS exposure. Ctsw (Cathepsin W), encoding a papain-like cysteine proteinase that is specifically expressed in natural killer cells (NK) and cytotoxic T cells, showed increased expression in CS-exposed *Hhip*^*+/-*^ mice compared to *Hhip*^*+/+*^ mice.

### HHIP haploinsufficiency led to activation of CD8+ T cells

**Figure 3:**
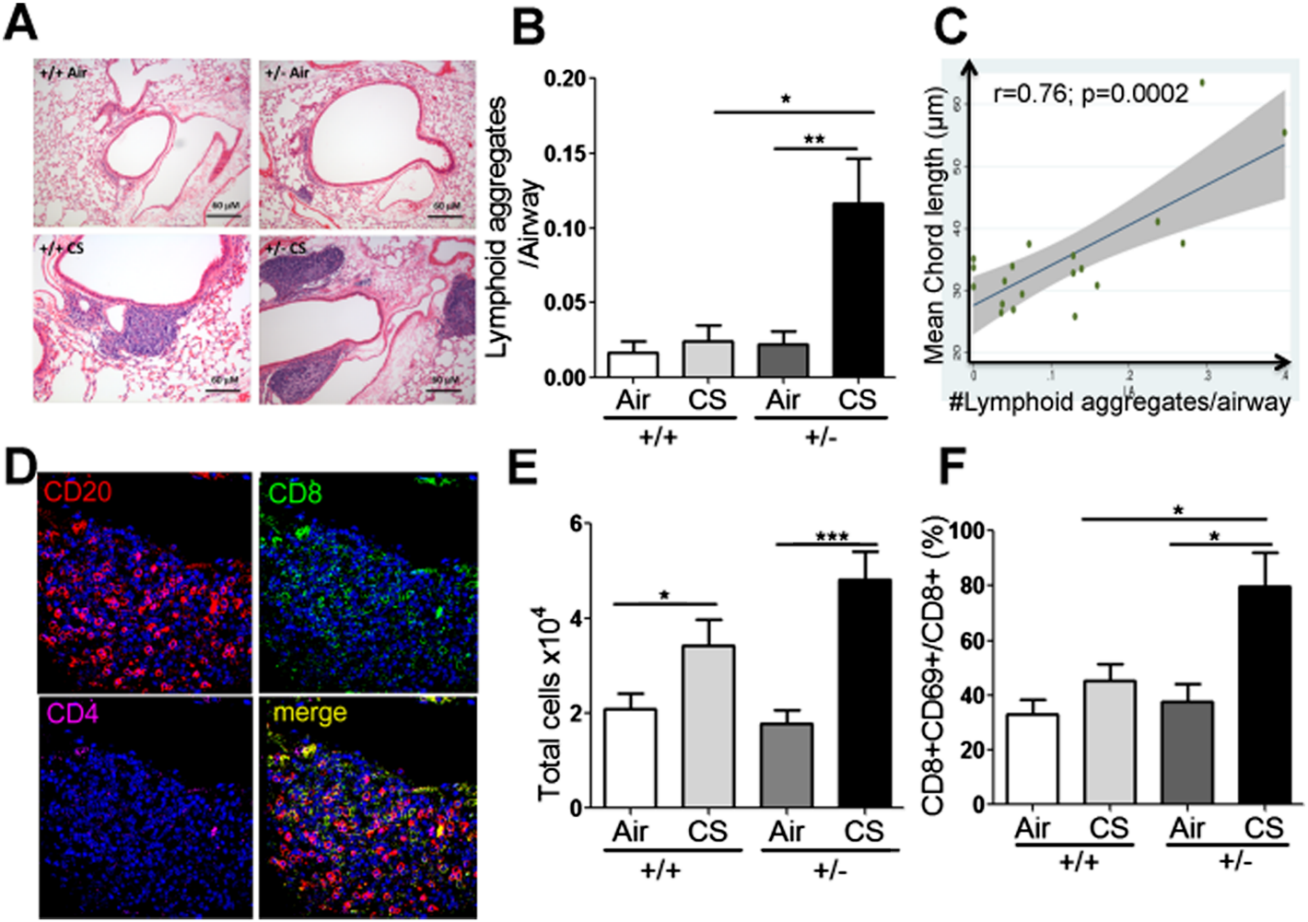
**Increased lymphocyte activation in the lungs of** ***Hhip*** ^***+/-***^ **mice exposed to cigarette smoke (CS). (A)** Representative Hematoxylin and Eosin staining images of lymphocyte aggregates around airways in murine lungs exposed to 6 months of air or CS. **(B)** Quantification of the number of lymphoid aggregates per airway (mean internal diameter of 200 to 1,000 μm) in (A) +/+: *Hhip* ^*+/+*^ mice; +/-, *Hhip* ^*+/-*^ mice. Data are mean ± SEM from >8 mice per group. **(C)** Correlation of the number of lymphoid aggregates per airway (X-axis) with the airspace size (mean chord length, MCL, Y-axis) of *Hhip* ^*+/-*^ mice exposed to CS for 6 months. Pearson correlation coefficients (r) and statistical significance (p) of the correlation coefficient are shown in the figure. **(D)** Representative triple immunostaining for CD20, CD4, and CD8 in a lymphocyte aggregate in a lung section from a Hhip^+/-^ mouse exposed to CS for 6 months. **(E)** Total leukocyte counts in bronchoalveolar lavage samples from *Hhip* ^*+/+*^ and *Hhip* ^*+/-*^ mice exposed to CS for 2 months. Data are means ± SEM from 10 to 11 mice per group. **(F)** Percentage of the CD8 + CD69+ T cells among total CD8+ T cells in lungs after enzymatic digestion from *Hhip* ^*+/-*^ and *Hhip* ^*+/+*^ mice exposed to CS for 2 months quantified using immunostaining and flow cytometry. Data are mean ± SEM; 3 to 6 mice per group. *P <0.05, **P <0.01.

Consistent with increased expression of genes involved in lymphocyte activation in *Hhip*^*+/-*^ mice exposed to CS, we also observed significantly increased numbers of lymphoid aggregates in *Hhip*^*+/-*^ mice exposed to CS chronically (Figure 3A, B). Similar to human COPD lungs [27], the presence of lymphoid aggregates near airways is strongly correlated with airspace size in CS-exposed *Hhip*^*+/-*^ mice (Figure 3C) but not *Hhip*^*+/+*^ mice (Additional file 1: Figure S4). We also performed triple staining with antibodies recognizing CD20 (a marker of B cells), CD8 and CD4 (markers of CD8+ and CD4+ T cells, respectively) in lung sections. As shown in Figure 3D, these peri-airway structures are indeed lymphoid aggregates composed of B cells and T cells (mainly CD8+ T cells along with a few CD4+ T cells). Given that CD8+ T lymphocyte activation has been shown to be required for CS-induced emphysema development in a murine model [28], we set out to quantify the number of CD8+ T cells in these four groups of mice using immunohistochemistry. Consistent with the previous report [28], we observed increased number of CD8+ T cells in *Hhip*^*+/+*^ mice exposed chronically to CS. More importantly, this CS-induced CD8+ T infiltration in the alveolar space is significantly enhanced in sections of lungs from Hhip^+/-^ mice (Additional file 1: Figure S5A).

Furthermore, a T lymphocyte-related cytokine, IL-2 [29], was also significantly increased in lung tissues of *Hhip*^*+/-*^ mice compared to *Hhip*^*+/+*^ mice, despite the lack of CS-induced increase in IL-2 levels in *Hhip*^*+/+*^ mice (Additional file 1: Figure S5B, two-way ANOVA analysis, *P* <0.05). Interferon gamma (IFN-γ) also showed a trend toward increased levels in the lungs of *Hhip*^*+/-*^ mice compared to *Hhip*^*+/+*^ mice after CS exposure (Additional file 1: Figure S5C). These changes in lung cytokine levels suggested a genotype-dependent response to CS in *Hhip*^*+/-*^ mice compared to *Hhip*^*+/+*^ mice.

In addition to chronic CS exposure, we also compared lung inflammatory responses in bronchoalvoelar lavage (BAL) samples from *Hhip*^*+/-*^ versus *Hhip*^*+/+*^ mice exposed to CS for 2 months. As expected, total cells in BAL samples increased in *Hhip*^*+/+*^ and *Hhip*^*+/-*^ mice exposed to 2 months of CS (Figure 3E) but total BAL cell counts were comparable in CS-exposed *Hhip*^*+/-*^ and CS-exposed *Hhip*^*+/+*^ mice. More importantly, the percentage of activated cytotoxic T cells, represented by positive staining for both CD8 and CD69, significantly increased in enzymatically digested lung samples from CS-exposed *Hhip*^*+/-*^ mice compared with those in CS-exposed *Hhip*^*+/+*^ mice as detected by flow cytometry (Figure 3F). In contrast, the percentage of activated CD4+ T cells increased in the lungs after CS exposure in both *Hhip*^*+/+*^ and *Hhip*^*+/-*^ mice, and CS-exposed *Hhip*^*+/-*^ mice had a similar percentage of activated CD4+ T cells in their lungs as CS-exposed *Hhip*^*+/+*^ mice (Additional file 1: Figure S5D), indicating that HHIP haploinsufficiency had less effect on the CS-induced activation of CD4+ T cells in murine lungs.

### Application of network models in mice exposed chronically to cigarette smoke

To interrogate potential regulatory mechanisms that differed in *Hhip*^*+/-*^ vs. *Hhip*^*+/+*^ mice and infer molecular driving factors that may contribute to the murine phenotypes described above, we constructed network models from microarray gene expression data using the PANDA (Passing Attributes between Networks for Data Assimilation) method [18]. PANDA integrates multiple sources of information, including protein-protein interaction, gene expression, and sequence motif data, to construct genome-wide, condition-specific regulatory networks. PANDA starts with a ‘prior’ map of potential transcription factor (TF)-gene interactions, often estimated by scanning the promoter regions of genes for the binding sites of TFs with known position-weight matrices. It then integrates this information with gene expression and protein-protein interaction data, using a message-passing approach to iteratively update the given initial map by evaluating (1) the similarity between gene co-expression and TF-targeting patterns as well as (2) the similarity between TF-TF (protein) interactions and input gene regulatory patterns. The result is a regulatory network estimate consistent with all sources of input data.

**Figure 4:**
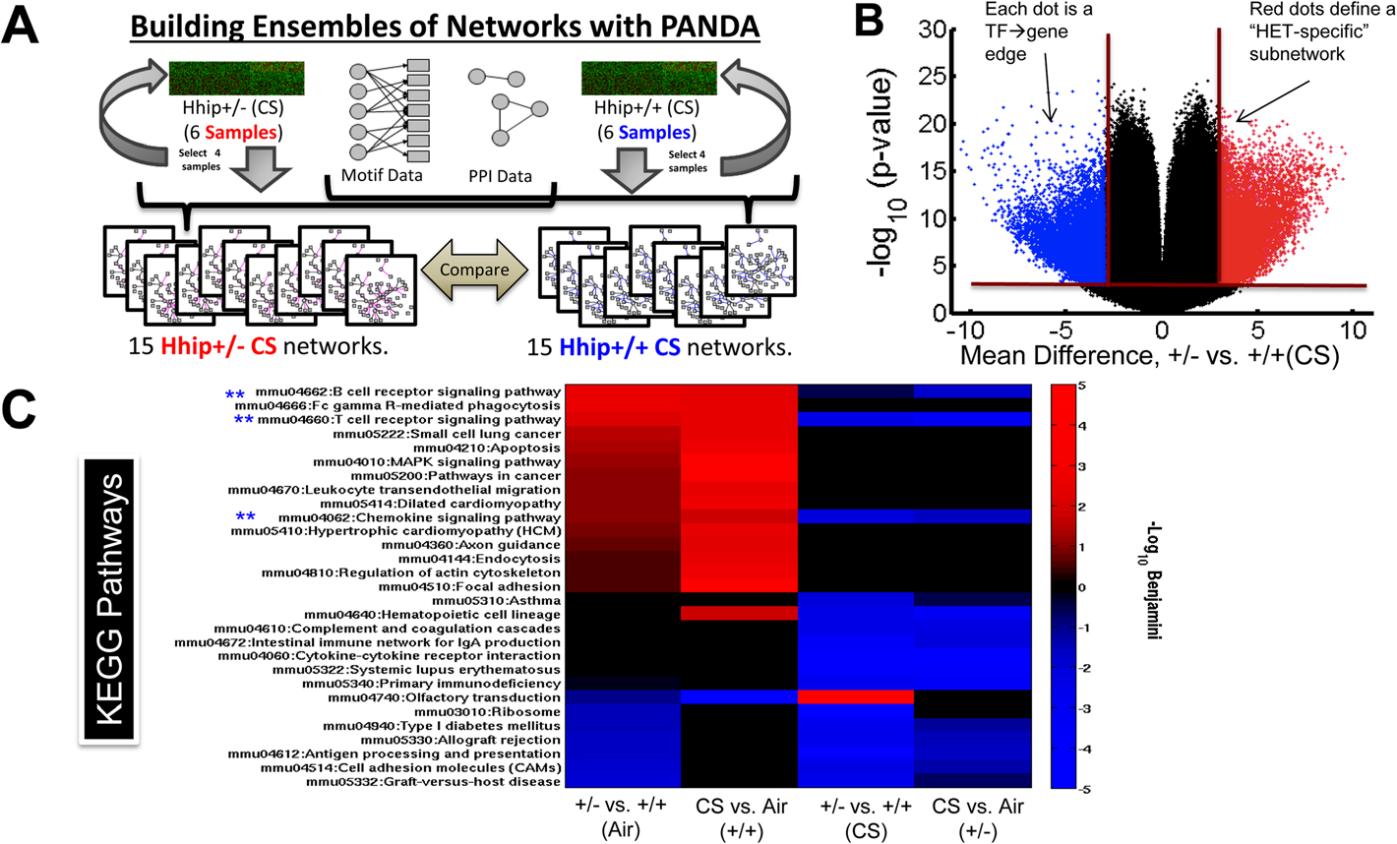
**Network analysis applying the ‘PANDA’ method to** ***Hhip*** ^***+/-***^ **mice vs.** ***Hhip*** ^***+/+***^ **mice treated with either room air (Air) or cigarette smoke (CS) for 6 months. (A)** Diagram to illustrate building networks using PANDA to combine transcription factor motif and physical protein interaction data with gene expression data from each group using a jack-knife subsampling method. **(B)** A volcano plot illustrating the selection of subnetworks by choosing high-probability edges specific to either the *Hhip* ^*+/-*^-CS (red) or *Hhip* ^*+/+*^-CS (blue) model. Red lines illustrate the thresholds used to select these edges, which were based on both the difference in average edge-weight (vertical lines) as well as *P* value significance (horizontal line). **(C)** Enriched KEGG Pathways (false discovery rate (FDR) <0.01) associated with at least one of eight distinct subnetworks, defined by four different comparisons (each column). FDR statistical significance is shown as a color with red colors representing more significant enrichment in subnetwork 1 (that is, *Hhip* ^*+/-*^ *-*CS in column 3) and blue colors representing more significant enrichment in subnetwork 2 (that is, *Hhip* ^*+/+*^-CS in column 3). Blue stars indicate lymphocyte activation pathway.

We applied PANDA to reconstruct sets of networks for the *Hhip*^*+/+*^-air, *Hhip*^*+/+*^-CS, *Hhip*^*+/-*^*-*air and *Hhip*^*+/-*^*-*CS groups (Figure 4A). To do this, we employed a ‘jack-knife’ technique to sample, without replacement, multiple times from each set of input expression arrays. This resulted in a set of networks for each condition of interest. For each set, we averaged the estimated weights (scores) for each potential interaction (or ‘edge’, representing transcriptional regulation between a transcription factor and a target gene). We also compared the distribution of weights for each interaction between network sets to quantify uncertainty in the predictions by *P* values. We used these two values to identify edges significantly different between each pair of conditions (absolute difference in average-weight >3 and *P* <0.001 based on an unpaired t-test comparing the distributions) (Figure 4B); the sets of edges that met these criteria define condition-specific subnetworks. The size of these subnetworks, based on the number of edges identified in each comparison, as well as the number of transcription factors and target genes associated with each subnetwork, is indicated in Additional file 2: Table S5. From these subnetworks, we set out to identify: (1) the genes that are differentially targeted under these four conditions; and (2) the TFs that differentially regulate genes under these four conditions. We focused on the comparison between CS-exposed *Hhip*^*+/-*^ and *Hhip*^*+/+*^ mice.

In PANDA networks, HHIP is predicted to be connected/regulated by 32 TFs with a putative binding site in the promoter of HHIP (Additional file 1: Figure S6). We then assessed significantly differentially targeted genes in each identified subnetwork by KEGG pathway analysis. The T cell receptor signaling pathways and chemokine signaling pathways were identified to be differentially regulated when comparing *Hhip*^*+/-*^ vs. *Hhip*^*+/+*^ mice either exposed to air or CS (indicated by blue stars in Figure 4C).

**Figure 5:**
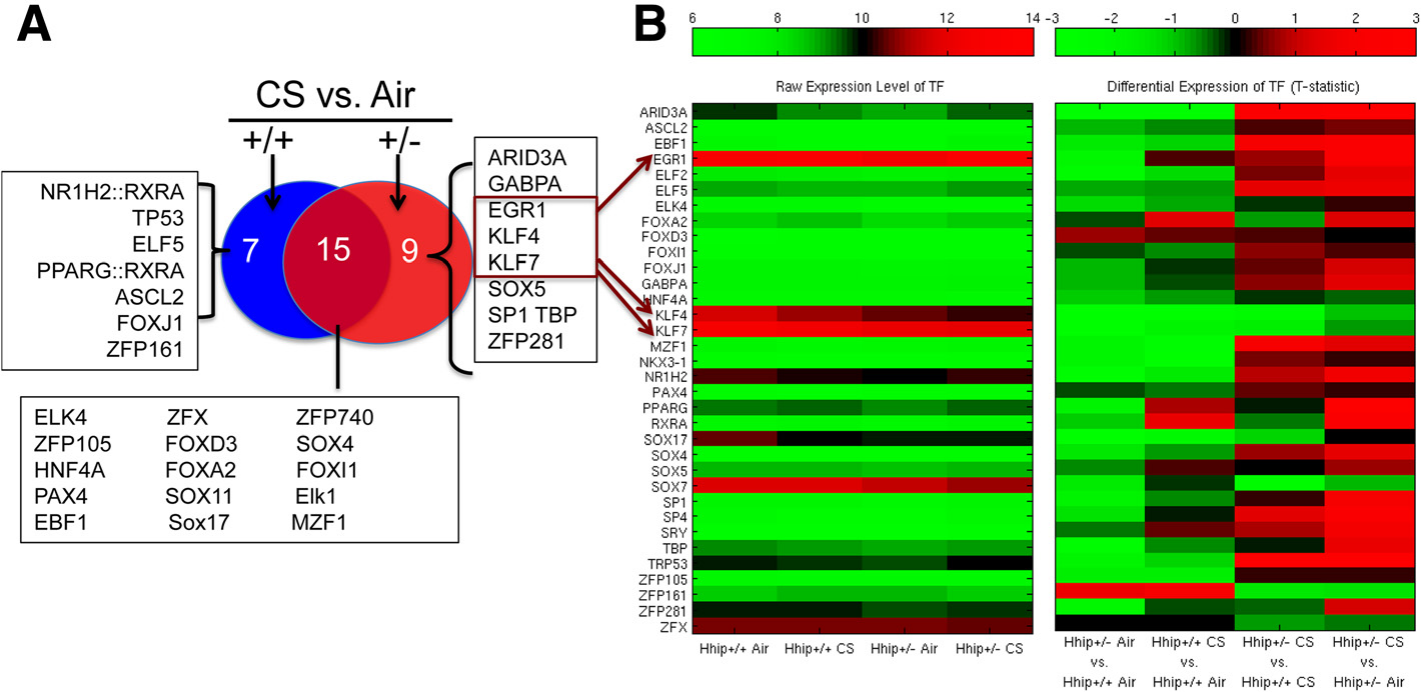
**Transcription factors identified from Subnetworks in PANDA analysis. (A)** Transcription factors (TFs) that govern subnetworks in *Hhip* ^*+/-*^ mice and *Hhip* ^*+/+*^ mice under CS vs. Air treatment. **(B)** Expression of all TFs identified from four subnetworks in murine lungs based on microarray data. Red arrows indicate top three highly expressed TFs in murine lungs that govern subnetworks in *Hhip* ^*+/-*^ mice and *Hhip* ^*+/+*^ mice under CS vs. Air treatment.

Furthermore, from TFs represented in these regulatory subnetworks (Additional file 2: Table S5), we generated a list of differentially regulating TFs based on significant changes in their associated edges (see details in Methods) between the subnetworks (Additional file 2: Tables S6 to S9). For TFs, we also determined their expression levels in the four experimental groups as well as the pairwise differential expression between the groups (using an unpaired t-test) (Figure 5A). Nine TFs were identified to specifically govern gene regulation in *Hhip*^*+/-*^ but not in *Hhip*^*+/+*^ under treatment of CS (Additional file 2: Tables S8 and S9). Among them, the top expressed TFs in murine lungs are Egr1 (early growth response protein 1), Klf4 (krüppel-like factor 4), and Klf7 (krüppel-like factor 7) (Figure 5B). Interestingly, Klf4, known to be important for T lymphocyte proliferation and activation [30], not only has differential connectivity in *Hhip*^*+/-*^-CS vs*. Hhip*^*+/+*^*-*CS*,* as revealed by PANDA analysis, it also demonstrated significantly reduced expression in *Hhip*^*+/-*^ murine lungs exposed to CS when compared to *Hhip*^*+/+*^ murine lungs exposed to CS (Additional file 1: Figure S7), suggesting a plausible mechanism by which *Hhip*^*+/-*^ mice enhanced lymphocyte activation after CS exposure.

Recent genome-wide association studies (GWAS) of COPD have identified several genomic regions that are clearly associated with COPD susceptibility; however, the biological mechanisms by which these genetic loci influence COPD remain undefined. Within one of the most well established COPD GWAS loci upstream from the *HHIP* gene on chromosome 4q31, we have previously identified a functional variant that exerts long-range regulation of the *HHIP* gene [7]. We have now extended those studies by exposing *Hhip*^*+/-*^ mice to chronic CS to model the biological impact of *HHIP* haploinsufficiency in CS-induced emphysema. We demonstrated that *HHIP* haploinsufficiency exaggerated CS-induced airspace enlargement. Gene expression profiling was used to investigate the molecular basis for the observed lung morphological and functional changes in *Hhip* haploinsufficient mice with chronic smoke exposure. Using PANDA, a recently developed network modeling approach, we characterized transcriptional regulatory networks that contribute to the CS-induced alterations in the *Hhip*^*+/-*^ murine lung. Among 15 identified TFs that control differential regulation in *Hhip*^*+/-*^*-*CS mice compared with *Hhip*^*+/+*^*-*CS mice, the top highly-expressed TFs in murine lungs include Klf4, which also showed reduced expression in lungs of CS-exposed *Hhip*^*+/-*^ mice compared to *Hhip*^*+/+*^ mice.

Functional interpretation of GWAS candidate regions/genes in complex diseases has been challenging in human genetics [31]. Given the early post-natal lethality in homozygous *Hhip* knockout mice and the moderate effects of the previously identified human functional variant on expression of *HHIP* [7], we evaluated a heterozygous murine model to dissect the function of this gene in a disease-relevant murine model. It is not unprecedented to observe significant phenotypic changes in heterozygous mice with deficiency of genes that are crucial for morphogenesis, such as *BECN1* [32], *TSC2* [33], and *NPM1* [34] - especially when tissue injury is induced in mice.

Increased lymphoid aggregates in human COPD lung samples were reported 10 years ago to be associated with progression of COPD [27]. We also observed similar lymphoid aggregates formed around airways in CS-exposed mice, and the number of these lymphoid aggregates significantly increased in *Hhip*^*+/-*^ mice exposed to CS. The presence of lymphoid aggregates was highly correlated with airspace enlargement in Hhip^+/-^ mice exposed to CS (Figure 3C), suggesting dysregulated immune responses in *Hhip*^*+/-*^ mice associated with the severity of airspace enlargement. Furthermore, subsequent FACS analysis demonstrated increased CD8+ T cell activation in *Hhip*^*+/-*^ mice exposed to 2 months of CS. Thus, CD8+ T cells may represent the major type of activated lymphocytes in *Hhip*^*+/-*^ mice exposed to CS, which is consistent with findings in human COPD lungs [27]. However, more in-depth investigations are needed to assess the involvement of B cell signaling pathways, as suggested by the pathway analysis based on gene-by-treatment interaction genes (Additional file 2: Table S2B).

In addition to airspace enlargement, we also observed increased lung compliance and reduced tissue elastance in Hhip^+/-^ mice exposed to CS (Figure 1B). However, we observed no significant changes of lung compliance and tissue elastance in wild type mice exposed to 6 months of chronic CS, similar to a previous study that failed to detect any significant differences in lung compliance between C57BL/6 wild-type mice exposed to air and smoke for 6 months [35]. The most likely reason for our inability to detect a CS-induced change in lung compliance in wild-type mice is that the emphysema that develops in this murine strain is relatively mild in severity. However, significant increases in lung compliance that we observed in CS-exposed *Hhip*^*+/-*^ mice when compared with CS-exposed *Hhip*^*+/+*^ mice are consistent with the increase in distal airspace size that we detected in CS-exposed *Hhip*^*+/-*^ mice. Thus, lung function changes in CS-exposed *Hhip*^*+/-*^ mice strongly supported that haploinsufficiency of *Hhip* increased the severity of CS-induced emphysema that develops in C57BL/6 strain mice.

Recent studies in murine models [36-38] and human samples [39] strongly supported pathological roles of activation of T cells in CS-induced emphysema development. Although similar contributions of CD4+ T helper or CD8+ T cytotoxic cells to airspace enlargement were observed in various CS-induced emphysema murine models [36-38], we observed significantly enhanced CD8+ T cell activation in *Hhip*^*+/-*^ mice that can produce deleterious effects leading to emphysema progression in both humans [27,40] and mice [28] due to recruitment of NK cells. In addition, activated CD8+ T cells in CS-exposed lungs release cytotoxic proteins, including perforin and granzyme, that promote cellular protein degradation and cleavage, leading to apoptotic alveolar cell death in CS-exposed lungs [40-42], thus contributing to emphysematous destruction in lungs.

Our network analysis, based on the PANDA method, provided novel gene regulatory insights into *Hhip* haploinsufficiency-imposed signaling rewiring in a systematic way. Among TFs that uniquely regulate the pulmonary response of *Hhip*^*+/-*^ mice to CS, Klf4 not only showed differential connectivity but also was significantly reduced in expression in *Hhip*^*+/-*^ murine lungs. Klf4 is important in maintaining T lymphocyte homeostasis as: (1) reduced expression of Klf4 is required for lineage commitment of T lymphocytes [43]; and (2) Klf4 directly regulates thymocyte proliferation [30]. More interestingly, the proliferation and homing of CD8+ T cells is controlled through a transcriptional regulation axis of Elf4-Klf4 and Elf4-Klf2 [44], which provides one plausible mechanism by which *HHIP* haploinsufficiency modulates lymphocyte activation through Klf4 signaling. This altered Klf4 signaling may be caused by either reduced expression of Klf4 in *Hhip*^*+/-*^ mice (Additional file 1: Figure S7) or differential targets of Klf4 in *Hhip*^*+/-*^ mice compared with *Hhip*^*+/+*^ mice in response to chronic CS exposure (Figure 5A). Additionally, a recent report showed that activation of the Hedgehog pathway in CD8+ T cells controlled killing function of cytotoxic T cells by polarizing the cytoskeleton to facilitate delivery of cytotoxic granules to the targets of cytotoxic T cells [45]. This also suggests that the Hedgehog pathway may contribute to the enhanced CD8+ T cell function in *Hhip*^*+/-*^ mice through a similar mechanism [45].

Together, our results indicate that after chronic CS exposure, Hhip haploinsufficient mice developed increased emphysema that was related to increased CD8+ T lymphocyte activation and differential Klf4 signaling in *Hhip*^*+/-*^ mice. In the future, additional comprehensive immunological experiments are needed to determine whether and how HHIP regulates lymphocyte proliferation, differentiation, and/or cytotoxic killing through Klf4 signaling and/or the Hedgehog pathway. Furthermore, whether Klf4 signaling and the Hedgehog pathway cooperate to modulate activation of naïve CD8+ T cells needs additional investigation.

There are several limitations to our study. First, the *HHIP* gene locus has also been associated with lung function in general population samples [46,47]. However, in our current system, no significant differences in lung morphology (Figure 1) between Air-exposed *Hhip*^*+/-*^*-*mice and *Hhip*^*+/+*^*-*mice were observed. This may be linked to the relatively modest reduction in *HHIP* expression in *Hhip*^*+/-*^ mice which may have been insufficient to result in significant lung function changes at the single time point studied here (8 months of age). Additional investigations are needed to explore how HHIP regulates lung function in non-smokers and whether there are common mechanisms shared between HHIP locus-regulated lung function and susceptibility to CS-induced emphysema. Second, transcription factors in the Hedgehog pathway, Gli1, Gli2, and Gli3, were not included in the murine motif database when we built PANDA networks. As a larger number of TFs are included in the existing motif database, our future network modeling studies using the PANDA method may reveal additional insights into the activities of Hhip in regulating CS-induced lung effects in mice. Third, we also attempted to compare gene expression changes caused by *Hhip* haploinsufficiency in murine lungs (*Hhip*^*+/-*^*-*air vs. *Hhip*^*+/+*^*-*air) with our previous microarray analysis in an airway epithelial cell line (Beas-2B cells), treated with either non-targeting shRNA controls or HHIP shRNAs [18]. No overlapping genes were identified (data not shown). This could be due to sample type differences (whole murine lung vs. a single human cell line), differential reductions in *HHIP* expression (approximately 33% vs. >70% reductions at mRNA level, respectively), or timing of sample collection (total lung lysates from mice at 8 months of age vs. cultured human cell lines). Lastly, we did not observe significantly increased lung levels of IL-2 and IFN-γ in wild type C57BL/6 mice exposed to 6 months of CS. Our results are consistent with a previous report showing that the C57BL/6 murine strain was a mildly susceptible strain with moderate emphysema development without any increases in lung IFN-γ levels after 6 months of CS exposure [35]. However, under the same CS exposure condition, we observed a trend toward increased IFN-γ in Hhip^+/-^ mice, suggesting the possibility of genotype-specific CD8+ T activation after CS exposure.

## Conclusions

In summary, combined with our previous finding that a COPD risk allele was associated with reduced enhancer activity for *HHIP* [7], our murine model has: (1) successfully demonstrated that modest reductions in *Hhip* gene expression, similar to smokers carrying *HHIP* COPD GWAS risk-alleles, led to increased susceptibility to CS-induced emphysema in *Hhip*^*+/-*^ mice; (2) shown that *HHIP* modulates lymphocyte activation especially in CD8+ T cells; (3) characterized networks with genes and TFs that are differentially regulated in murine lungs responsive to either CS treatment or haploinsufficiency of *Hhip*; and (4) revealed roles of *Hhip* in maintaining lung homeostasis that is disrupted in the haploinsufficiency state in the adult murine lung in addition to its known roles in embryonic lung development (identified using a loss-of-function approach) [10]. Identification of strategies to correct alterations in network rewiring in *Hhip*^*+/-*^ mice could eventually facilitate the development of novel therapies for human COPD.

## Additional files

Additional file 1: Figure S1. RT-PCR measurements of HHIP expression in murine lungs. **Figure S2.** Gene expression of Hedgehog pathway components in murine lungs. **Figure S3.** Differential lung tissue gene expression related to Hhip genotype and cigarette smoke exposure based on microarray analysis using gene probesets. **Figure S4.** Correlation of the number of lymphoid aggregates with airspace size in murine lungs. **Figure S5.** Differential T cell signaling in murine lungs from Hhip^+/-^ mice exposed to 6 months **(A-C)** or 2 months **(D)** of cigarette smoke compared with Hhip^+/+^ mice. **Figure S6.** Transcription factors connected with HHIP in the subnetworks generated by PANDA analysis. **Figure S7.** Expression of Klf4 in Hhip^+/-^ murine lungs revealed by microarray and RT-PCR analysis. Additional file 2: Table S1A. Number of differentially expressed gene probes (*P* adj <0.05) in *Hhip* ^*+/-*^ and *Hhip* ^*+/+*^ mice exposed to either room air (Air) or cigarette smoke (CS) for 6 months. **Table S2B.** Top 10 GO- term pathways out of 81 significant pathways (FDR <0.05) are listed here ranked by FDR. **Table S3.** The List (A) and GeneMANIA functional annotation (B) of 41 genes that showed statistically significant gene-by-treatment interaction (www.genemania.org) [7] and also were differentially expressed in *Hhip* ^*+/-*^ *-*CS versus *Hhip* ^*+/+*^ *-*CS mice using the default list of networks and weighting. **Table S3A.** List of 41 genes ranked based on adjusted *P* value for interaction term. T statistics in the second column indicate statistical analysis on expression comparisons between *Hhip* ^*+/-*^ *-*CS vs. *Hhip* ^*+/+*^ *-* CS mice. Positive t values mean increased expression in *Hhip* ^*+/-*^ *-*CS and negative t values mean decreased expression in *Hhip* ^*+/+*^ *-*CS mice. **Table S3B.** All significant pathways enriched in 41 genes were listed here ranked by FDR. **Table S4.** Summary of real-time RT-PCR validation of selected differentially expressed genes that were identified by microarray analysis in four groups of mice. Fold changes were calculated based on average relative expression of each gene in six mice from each group. GAPDH was used as a reference gene for RT-PCR. All gene expression changes in RT-PCR are consistent with microarray analysis. * Indicates *P* <0.05; ** indicates *P* <0.01 based on unpaired *t* test. **Table S5.** Characteristics of subnetworks in four pair-wise comparisons based on PANDA analysis. **Table S6.** Transcription factors (TFs) regulating differential subnetworks in *Hhip* ^*+/-*^-Air and *Hhip* ^*+/+*^ *-*Air subnetworks identified by PANDA analysis. **Table S7.** Transcription factors (TFs) in *Hhip* ^*+/-*^ *-*CS and *Hhip* ^*+/+*^ *-*CS subnetworks identified by PANDA analysis. **Table S8.** Transcription factors (TFs) regulating differential subnetworks in *Hhip* ^*+/-*^-CS and *Hhip* ^*+/-*^-Air subnetworks identified by PANDA analysis. **Table S9.** Transcription factors (TFs) in *Hhip* ^*+/+*^ *-*CS and *Hhip* ^*+/+*^ *-*Air identified by PANDA analysis. Additional file 3: Table S1B. The list of genes differentially expressed by pair-wise comparisons among four groups of mice. Additional file 4: Table S2. List of 266 genes that showed statistically significant gene-by-treatment interaction (http://www.genemania.org/) [7] using the default list of networks and weighting.
