# Supplementary material for: Haploinsufficiency of Hedgehog interacting protein causes increased emphysema induced by cigarette smoke through network rewiring
Source: Genome Med. 2015 Feb 14;7(1):12. doi: 10.1186/s13073-015-0137-3 (PMC4355149; doi:10.1186/s13073-015-0137-3)
Supplement: Additional file 1: Figure S1. — RT-PCR measurements of HHIP expression in murine lungs. Figure S2. Gene expression of Hedgehog pathway components in murine lungs. Figure S3. Differential lung tissue gene expression related to Hhip genotype and cigarette smoke exposure based on microarray analysis using gene probesets. Figure S4. Correlation of the number of lymphoid aggregates with airspace size in murine lungs. Figure S5. Differential T cell signaling in murine lungs from Hhip+/- mice exposed to 6 months (A-C) or 2 months (D) of cigarette smoke compared with Hhip+/+ mice. Figure S6. Transcription factors connected with HHIP in the subnetworks generated by PANDA analysis. Figure S7. Expression of Klf4 in Hhip+/- murine lungs revealed by microarray and RT-PCR analysis. [file 13073_2015_137_MOESM1_ESM.pdf]

Supplemental Figure 1: RT-PCR measurements of HHIP expression in murine lungs.

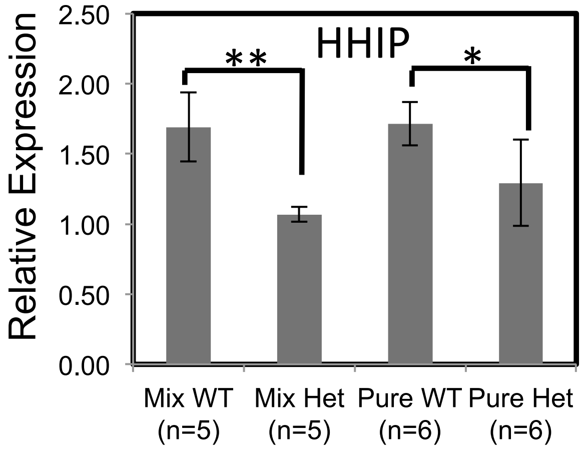

Supplemental Figure 1. Gene expression of *Hhip* in lung tissues from *Hhip*$^{+/-}$ mice in mixed and pure C57BL/6J backgrounds measured by real-time RT-PCR. GAPDH (glyceraldehyde 3-phosphate dehydrogenase) was used as a reference gene. *, p<0.05 and **, p<0.01. Means±SD are shown in each group.

Supplemental Figure 2. Gene expression of Hedgehog pathway components in murine lungs.

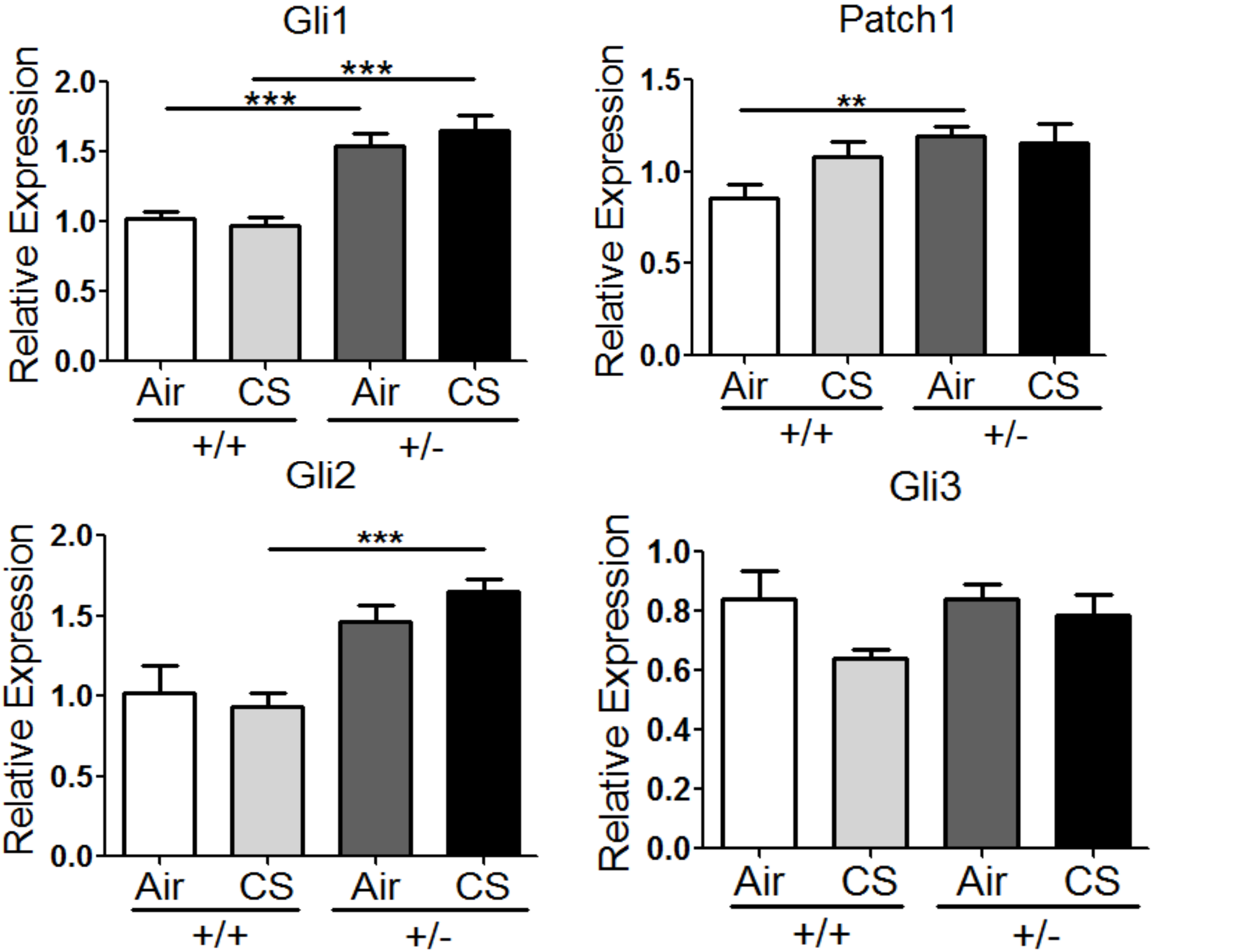

Supplemental Figure 2. Gene expression measurements of mouse *Gli1*, *Gli2, Gli3* and *Ptch1* in lung tissues from *Hhip*$^{+/-}$ (+/-) and *Hhip*$^{+/+}$ mice(+/+) exposed to air or CS for 6 months detected by real-time RT-PCR. GAPDH (Glyceraldehyde 3-phosphate dehydrogenase) was used as a reference gene. Means±SEM were from at least 6 mice in each group. ***, p<0.001; **, p<0.01.

Supplemental Figure 3: Differential lung tissue gene expression related to Hhip genotype and cigarette smoke exposure based on microarray analysis using gene probesets.

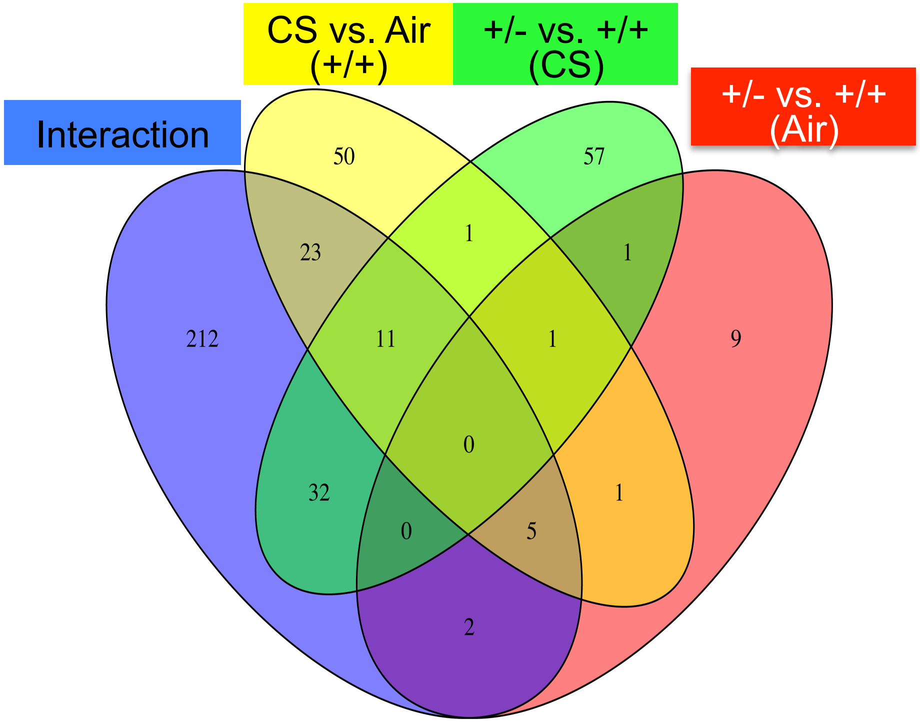

Supplemental Figure 3. Venn diagram shows comparisons of differentially expressed genes using probesets in *Hhip*$^{+/+}$-CS vs. *Hhip*$^{+/+}$-Air (yellow oval), *Hhip*$^{+/-}$-CS vs. *Hhip*$^{+/+}$-CS (green oval), *Hhip*$^{+/-}$-Air vs. *Hhip*$^{+/+}$-Air (red oval) and genes enriched based on interaction with smoke treatment (blue oval). +/+: *Hhip*$^{+/+}$; +/-: *Hhip*$^{+/-}$; CS: cigarette smoke.

Supplemental Figure 4: Correlation of the number of lymphoid aggregates with airspace size in murine lungs.

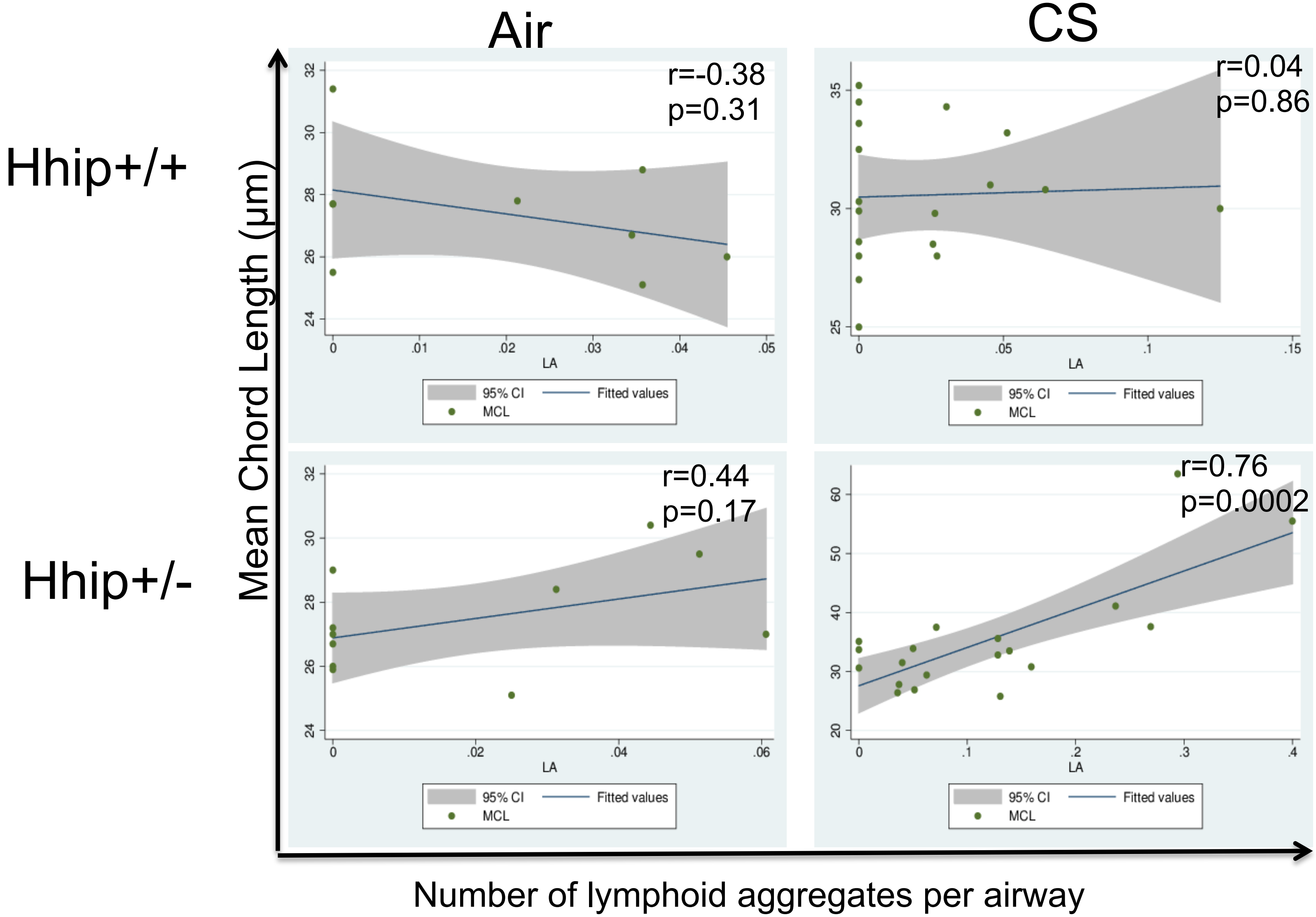

Supplemental Figure 4. Correlation of the number of lymphoid aggregates per airway (200-1000µm) with the airspace size (mean chord length, MCL) in *Hhip*$^{+/+}$ and *Hhip*$^{+/-}$ mice exposed to Air or cigarette smoke (CS) for 6 months. Pearson correlation coefficients (r) and statistical significance (p) of the correlation coefficient are shown in each graph. CS: cigarette smoke.

Supplemental Figure 5: Differential T cells signaling in murine lungs from Hhip+/- mice exposed to 6 months (A-C) o
2 months (D) of cigarette smoke compared with Hhip+/+ mice.

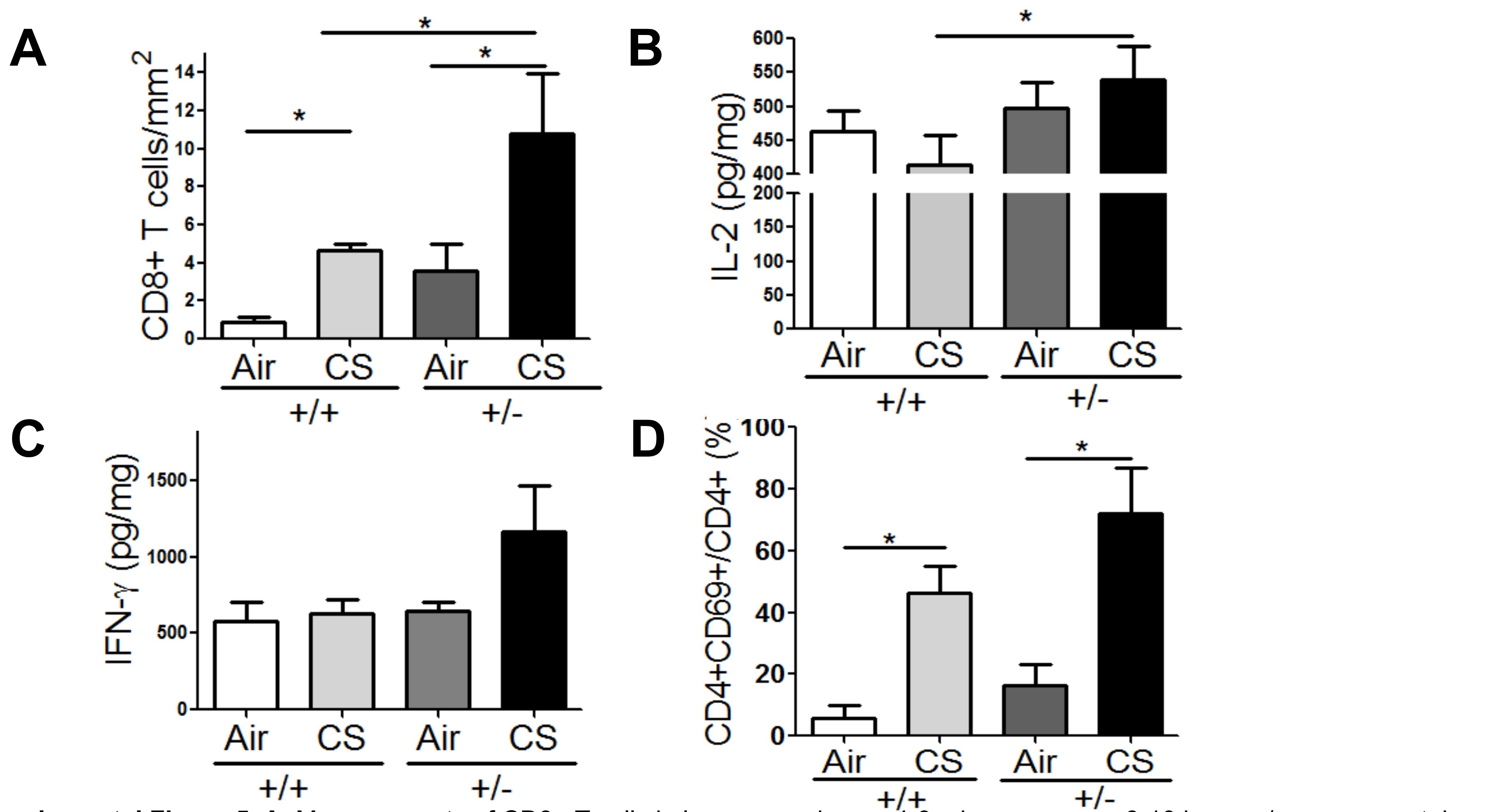

Supplemental Figure 5. A. Measurements of CD8+ T cells in lung parenchyma, 4-9 mice per group. 6-10 images/mouse were taken to quantify number of CD8+ T cells in the alveolar space. Results were expressed as the number of CD8+ T cells per alveolar space area. By Kruskal-Wallis test, we detected significant difference among groups ($p<0.001$). Results from Wilcoxon rank sum test for pair-wised comparisons are shown here. **B.** Measurements of IL-2 by ELISA in murine lungs from *Hhip*$^{+/+}$ and *Hhip*$^{+/-}$ mice exposed to 6 months of CS. Means±SEM are from 8-11 mice per group. **C.** Measurements of IFN-γ by ELISA in murine lungs from *Hhip*$^{+/+}$ and *Hhip*$^{+/-}$ mice exposed to 6 months of CS. Means±SEM are from 7-10 mice per group. A trend toward significance between CS-exposed *Hhip*$^{+/-}$ vs. *Hhip*$^{+/+}$ mice was detected by unpaired t test. **D.** Percentage of the CD4+CD69+ T cells among total CD4+ T cells in murine lungs from *Hhip*$^{+/-}$ and *Hhip*$^{+/+}$ mice exposed to CS for 2 months detected by flow cytometry. Means±SEM were from 3-6 mice per group. *, $p<0.05$, **, $p<0.01$. +/+: *Hhip*$^{+/+}$; +/-: *Hhip*$^{+/-}$; CS: cigarette smoke.

Supplemental Figure 6. Transcription factors connected with HHIP in the subnetworks generated by PANDA analysis.

	Hhip+/- vs Hhip+/+ (Air)	CS vs Air (Hhip+/+)	Hhip+/- vs Hhip+/+ (CS)	CS vs Air (Hhip+/-)
CREB1	0.445 (1.02e-07)	0.914 (2.97e-17)	-0.568 (1.21e-06)	-0.099 (3.29e-01)
EGR1	2.300 (8.52e-04)	2.629 (1.42e-04)	-0.140 (7.00e-01)	0.189 (6.32e-01)
ESR1	0.636 (1.42e-04)	0.881 (7.93e-07)	-0.335 (2.85e-03)	-0.089 (4.19e-01)
FOXI1	-0.336 (1.63e-01)	-1.212 (1.69e-05)	1.463 (2.59e-10)	0.587 (7.00e-04)
FOXQ1	-0.617 (3.13e-07)	-1.220 (1.67e-18)	0.931 (3.27e-09)	0.328 (1.83e-02)
KLF4	2.320 (1.36e-03)	2.676 (2.20e-04)	-0.229 (5.67e-01)	0.127 (7.70e-01)
MZF1_5-13	0.781 (6.69e-03)	0.756 (6.84e-03)	0.238 (9.55e-02)	0.212 (1.73e-01)
NF-KAPPAB	0.554 (2.49e-09)	0.878 (6.48e-14)	-0.516 (2.35e-10)	-0.193 (1.36e-03)
NKX3-2	-0.177 (2.63e-01)	0.181 (2.44e-01)	-0.359 (1.96e-04)	-0.001 (9.88e-01)
PLAG1	0.274 (3.63e-04)	0.777 (1.01e-13)	-0.648 (8.86e-13)	-0.145 (2.97e-02)
REL	0.514 (9.33e-09)	0.745 (3.33e-13)	-0.533 (1.03e-09)	-0.303 (7.18e-05)
RELA	0.388 (1.45e-10)	0.697 (1.14e-18)	-0.555 (6.04e-15)	-0.246 (3.39e-06)
SP1	2.379 (3.12e-03)	2.537 (1.45e-03)	0.073 (8.66e-01)	0.230 (6.18e-01)
TAL1::TCF3	-0.236 (3.84e-03)	-0.327 (2.55e-04)	0.178 (1.23e-02)	0.088 (1.74e-01)
ATF1	0.095 (3.89e-01)	0.587 (9.47e-06)	-0.569 (4.78e-07)	-0.077 (3.84e-01)
E2F2	1.045 (6.12e-09)	1.611 (2.10e-15)	-0.880 (2.70e-06)	-0.313 (7.26e-02)
E2F3	0.992 (1.60e-09)	1.628 (1.14e-17)	-0.996 (1.01e-07)	-0.360 (3.14e-02)
HMBOX1	-0.442 (3.90e-03)	-0.763 (5.46e-06)	0.245 (1.11e-02)	-0.076 (4.38e-01)
JUNDM2	0.056 (6.22e-01)	0.380 (2.26e-03)	-0.361 (8.95e-05)	-0.036 (6.39e-01)
KLF7	2.259 (7.43e-04)	2.929 (2.07e-05)	-0.594 (1.20e-01)	0.076 (8.53e-01)
OSR1	-0.306 (5.65e-04)	-0.302 (3.16e-03)	0.012 (8.86e-01)	0.015 (8.03e-01)
OSR2	-0.258 (1.70e-02)	-0.265 (3.07e-02)	-0.121 (1.80e-01)	-0.129 (6.59e-02)
PLAGL1	0.993 (3.47e-07)	1.408 (1.74e-11)	-0.527 (4.68e-04)	-0.113 (4.65e-01)
SMAD3	0.172 (1.70e-01)	0.104 (4.13e-01)	0.129 (6.83e-02)	0.061 (3.28e-01)
SP4	2.213 (4.52e-04)	2.863 (9.90e-06)	-0.697 (7.06e-02)	-0.046 (9.10e-01)
TCF2	-0.235 (2.00e-01)	-0.287 (1.09e-01)	-0.235 (1.80e-02)	-0.287 (1.08e-02)
TCFAP2E	1.044 (6.15e-06)	1.348 (2.40e-08)	-0.426 (3.90e-03)	-0.122 (4.25e-01)
ZBTB12	-0.151 (3.03e-01)	-0.108 (4.51e-01)	-0.278 (5.63e-03)	-0.234 (2.41e-02)
ZBTB3	0.326 (2.25e-03)	0.421 (1.49e-04)	-0.007 (9.06e-01)	0.089 (1.28e-01)
ZFP161	1.293 (2.34e-06)	1.939 (5.56e-11)	-0.961 (6.76e-05)	-0.316 (1.87e-01)
ZFP281	1.461 (5.37e-03)	1.385 (6.77e-03)	0.352 (1.85e-01)	0.276 (3.29e-01)
ZFP740	0.969 (1.26e-02)	1.011 (7.92e-03)	0.263 (1.92e-01)	0.305 (1.65e-01)

Supplemental Figure 7 Expression of Klf4 in *Hhip*$^{+/-}$ murine lungs revealed by microarray and RT-PCR.

A

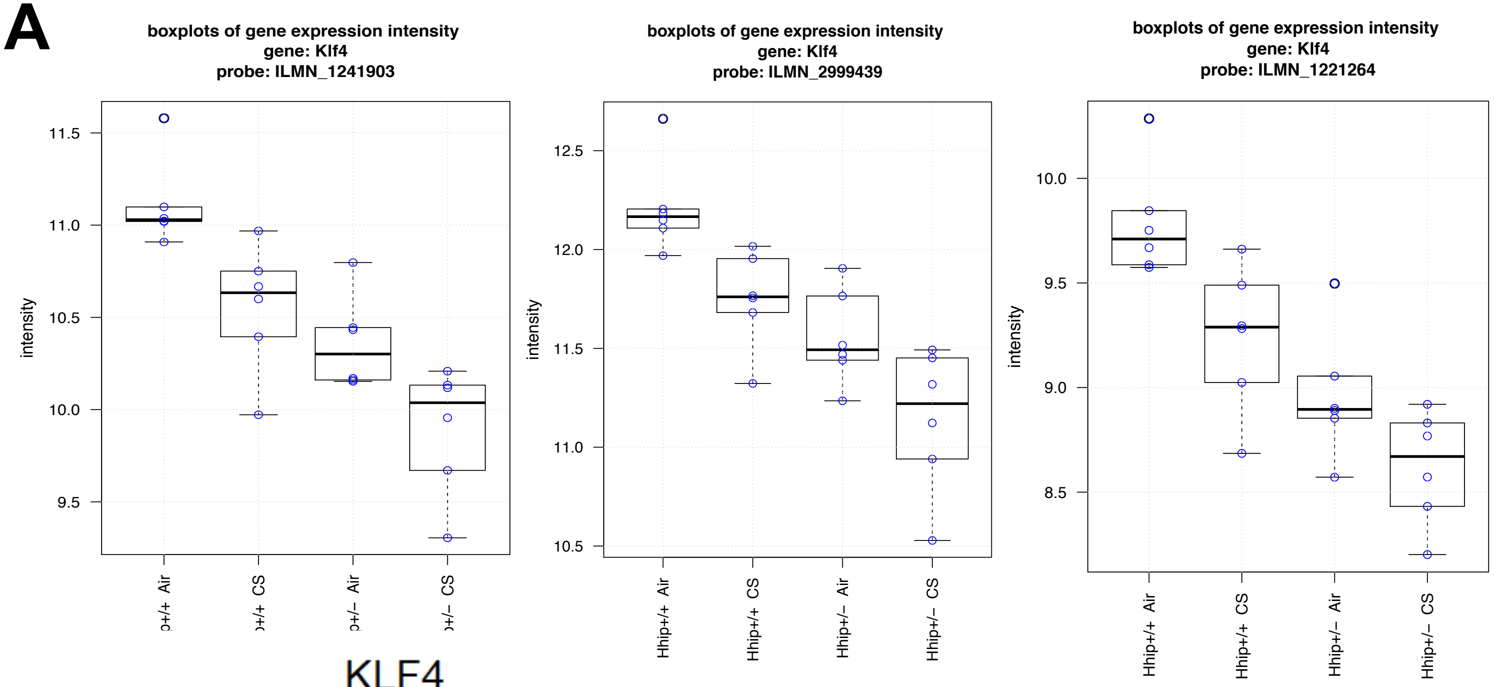

B

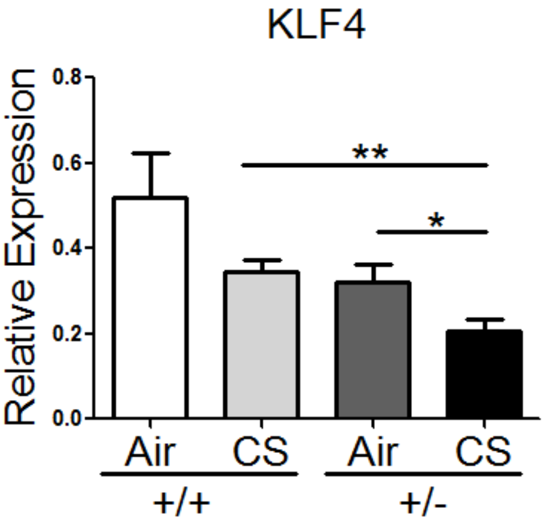

Supplemental Figure 7. Expression of *Klf4* in murine lungs from four groups of mice (N=6/group). A. Box plots for three probe sets targeting murine *Klf4* gene as revealed in microarray analysis and real-time RT-PCR (B) in murine lungs exposed to 6 months of cigarette smoke. Means±SEM are from 6 mice/group. Two-way ANOVA analysis showed significant difference between two genotypes and between air and CS treatment. (p<0.01). **, p<0.01. +/+: *Hhip*$^{+/+}$; +/-: *Hhip*$^{+/-}$; CS: cigarette smoke.
