# Supplementary material for: Haploinsufficiency of Hedgehog interacting protein causes increased emphysema induced by cigarette smoke through network rewiring
Source: Genome Med. 2015 Feb 14;7(1):12. doi: 10.1186/s13073-015-0137-3 (PMC4355149; doi:10.1186/s13073-015-0137-3)
Supplement: Additional file 2: Table S1A. — Number of differentially expressed gene probes (P adj <0.05) in Hhip +/- and Hhip +/+ mice exposed to either room air (Air) or cigarette smoke (CS) for 6 months. Table S2B. Top 10 GO- term pathways out of 81 significant pathways (FDR <0.05) are listed here ranked by FDR. Table S3. The List (A) and GeneMANIA functional annotation (B) of 41 genes that showed statistically significant gene-by-treatment interaction (www.genemania.org) [7] and also were differentially expressed in Hhip +/- -CS versus Hhip +/+ -CS mice using the default list of networks and weighting. Table S3A. List of 41 genes ranked based on adjusted P value for interaction term. T statistics in the second column indicate statistical analysis on expression comparisons between Hhip +/- -CS vs. Hhip +/+ - CS mice. Positive t values mean increased expression in Hhip +/- -CS and negative t values mean decreased expression in Hhip +/+ -CS mice. Table S3B. All significant pathways enriched in 41 genes were listed here ranked by FDR. Table S4. Summary of real-time RT-PCR validation of selected differentially expressed genes that were identified by microarray analysis in four groups of mice. Fold changes were calculated based on average relative expression of each gene in six mice from each group. GAPDH was used as a reference gene for RT-PCR. All gene expression changes in RT-PCR are consistent with microarray analysis. * Indicates P <0.05; ** indicates P <0.01 based on unpaired t test. Table S5. Characteristics of subnetworks in four pair-wise comparisons based on PANDA analysis. Table S6. Transcription factors (TFs) regulating differential subnetworks in Hhip +/--Air and Hhip +/+ -Air subnetworks identified by PANDA analysis. Table S7. Transcription factors (TFs) in Hhip +/- -CS and Hhip +/+ -CS subnetworks identified by PANDA analysis. Table S8. Transcription factors (TFs) regulating differential subnetworks in Hhip +/--CS and Hhip +/--Air subnetworks identified by PANDA analysis. Table S9. Transcription f [file 13073_2015_137_MOESM2_ESM.doc]

Supplemental Table 1A. Number of differentially expressed gene probes (p adj<0.05) in *Hhip^+/-^* and *Hhip^+/+^* mice exposed to either room air (Air) or cigarette smoke (CS) for 6 months.

| Comparisons (6 mice/group) | Increased Expression | Decreased Expression | Total |
| --- | --- | --- | --- |
| *Hhip^+/+^*: CS vs. Air | 64 | 27 | 91 |
| *Hhip^+/-^*: CS. vs Air | 2 | 1 | 3 |
| *Hhip^+/-^*-Air vs. *Hhip^+/+^*-Air | 10 | 9 | 19 |
| *Hhip^+/-^*-CS vs. *Hhip^+/+^*-CS | 47 | 56 | 103 |

Supplemental Table 1B. The list of genes differentially expressed by pair-wise comparisons among group groups of mice. See attached excel file.

Supplemental Table 2. List (A, see attached excel file) and GeneMANIA functional annotation (B) on 266 genes that showed statistically significant gene-by-treatment interaction (<http://www.genemania.org/>) using the default list of networks and weighting.

Supplemental Table 2B. Top 10 GO- term pathways out of 81 significant pathways (FDR<0.05) are listed here ranked by False Discovery Rate (FDR).

| Feature | FDR | Genes in network | Genes in genome |
| --- | --- | --- | --- |
| B cell activation | 1.31E-05 | 17 | 199 |
| reactive oxygen species metabolic process | 1.32E-05 | 14 | 132 |
| myeloid leukocyte activation | 2.17E-05 | 13 | 118 |
| B cell receptor signaling pathway | 2.49E-05 | 8 | 33 |
| positive regulation of immune response | 2.49E-05 | 19 | 292 |
| immune response-activating signal transduction | 3.19E-05 | 15 | 180 |
| antigen receptor-mediated signaling pathway | 3.19E-05 | 11 | 86 |
| immune response-regulating signaling pathway | 6.59E-05 | 15 | 194 |
| immune response-activating cell surface receptor signaling pathway | 1.11E-04 | 11 | 99 |
| activation of immune response | 1.42E-04 | 15 | 209 |

Supplemental Table 3. The List (A) and GeneMANIA functional annotation (B) of 41 genes that showed statistically significant gene-by-treatment interaction (<http://www.genemania.org/>) and also were differentially expressed in *Hhip^+/-^-*CS versus. *Hhip^+/+^-*CS mice using the default list of networks and weighting.

Supplemental Table 3A. List of 41 genes ranked based on adjusted p value for interaction term. T statistics in the second column indicate statistical analysis on expression comparisons between *Hhip^+/-^-*CS vs. *Hhip^+/+^-* CS mice. Positive t values mean increased expression in *Hhip^+/-^-*CS and negative t values mean decreased expression in *Hhip^+/+^-*CS mice.

| Symbol | t | P.Value | adj. P.Val | treatment.p.value | genotype.p.value |
| --- | --- | --- | --- | --- | --- |
| Arhgap4 | 6.2536706 | 2.74E-06 | 0.00466483 | 0.000114186 | 7.03E-07 |
| Cbr3 | -6.26381 | 2.68E-06 | 0.00466483 | 0.000205993 | 4.60E-08 |
| Ncf1 | 6.2761935 | 2.60E-06 | 0.00466483 | 5.44E-05 | 1.40E-05 |
| Prkcb | 6.6174625 | 1.20E-06 | 0.00466483 | 1.60E-05 | 2.51E-06 |
| Tmtc1 | -6.764452 | 8.61E-07 | 0.00466483 | 0.000112113 | 4.58E-05 |
| Lacc1 | 6.6618431 | 1.08E-06 | 0.00466483 | 0.000320338 | 4.46E-05 |
| Dennd1c | 6.1828574 | 3.23E-06 | 0.004877021 | 4.60E-05 | 2.34E-06 |
| Selplg | 6.1524458 | 3.46E-06 | 0.004877021 | 0.000256977 | 9.93E-06 |
| Hcst | 6.1111731 | 3.81E-06 | 0.004955304 | 2.28E-05 | 5.82E-06 |
| Cyp1a1 | -6.053761 | 4.35E-06 | 0.005346209 | 0.128639669 | 6.90E-08 |
| Pou2f2 | 5.5191083 | 1.53E-05 | 0.014086231 | 0.000524245 | 0.000466817 |
| Il2rg | 5.4680733 | 1.73E-05 | 0.015268725 | 0.00983532 | 0.00010647 |
| Dtna | -5.279324 | 2.71E-05 | 0.015370947 | 0.000431423 | 5.14E-06 |
| Fyb | 5.3419673 | 2.33E-05 | 0.015370947 | 0.00152057 | 0.000318216 |
| Lat2 | 5.3362863 | 2.36E-05 | 0.015370947 | 2.74E-05 | 1.18E-05 |
| Pip4k2a | 5.232858 | 3.03E-05 | 0.016309692 | 0.001189019 | 5.64E-05 |
| Ccdc88b | 5.1964794 | 3.31E-05 | 0.017004804 | 0.008517023 | 5.74E-05 |
| Krt80 | -5.135568 | 3.83E-05 | 0.017106207 | 0.001083105 | 6.14E-07 |
| Sash3 | 5.1436779 | 3.75E-05 | 0.017106207 | 0.000110051 | 1.04E-05 |
| Hspa4l | -4.983192 | 5.53E-05 | 0.018270443 | 0.007549392 | 8.27E-06 |
| Slc6a2 | -4.81733 | 8.26E-05 | 0.021100101 | 0.007718001 | 2.34E-06 |
| Pstpip1 | 4.7535726 | 9.65E-05 | 0.022468035 | 0.001083563 | 1.08E-05 |
| Ptpn18 | 4.6914512 | 0.00011223 | 0.025332491 | 0.002025098 | 8.87E-06 |
| Slc11a1 | 4.5475566 | 0.00015934 | 0.030125268 | 0.020070636 | 0.007927006 |
| Ctsw | 4.4833895 | 0.00018632 | 0.030323759 | 0.000608624 | 1.80E-05 |
| Haao | 4.5126666 | 0.00017348 | 0.030323759 | 0.001377847 | 1.80E-05 |
| Ikbkg | -4.483152 | 0.00018643 | 0.030323759 | 0.138993616 | 1.12E-05 |
| Coro2b | -4.471206 | 0.00019194 | 0.030546571 | 0.000760991 | 5.60E-06 |
| Kcnab2 | 4.4581159 | 0.00019817 | 0.030794539 | 0.000613893 | 2.50E-05 |
| Tinagl | -4.456471 | 0.00019897 | 0.030794539 | 2.26E-06 | 2.70E-07 |
| Kcne2 | -4.387685 | 0.00023533 | 0.032443766 | 2.92E-05 | 9.07E-06 |
| Ryr1 | 4.4110553 | 0.00022228 | 0.032443766 | 0.050120258 | 0.002400025 |
| Evi2b | 4.3130513 | 0.00028234 | 0.033498126 | 0.020000268 | 7.03E-06 |
| Cyp1b1 | -4.280129 | 0.00030597 | 0.034615165 | 6.48E-12 | 1.54E-05 |
| Itgb7 | 4.267998 | 0.00031516 | 0.035055868 | 0.000161602 | 1.05E-05 |
| Pxdc1 | -4.189646 | 0.00038156 | 0.03854557 | 2.57E-05 | 1.05E-05 |
| Wisp2 | 4.0803496 | 0.0004981 | 0.044692303 | 0.000596247 | 5.46E-08 |
| Arap1 | 4.0691303 | 0.00051191 | 0.045340768 | 0.000479431 | 0.000244179 |
| Klhl6 | 4.0529895 | 0.00053244 | 0.045829282 | 0.000494769 | 1.16E-05 |
| Ccdc67 | -2.054716 | 0.05200677 | 0.330739984 | 0.048437211 | 0.315081659 |
| Thap11 | -2.041477 | 0.05341076 | 0.33392755 | 0.158073627 | 0.078671537 |

Supplemental Table 3B. All significant pathways enriched in 41 genes were listed here ranked by FDR.

| Feature | FDR | Genes in network | Genes in genome |
| --- | --- | --- | --- |
| B cell receptor signaling pathway | 5.22E-04 | 5 | 33 |
| B cell activation | 5.54E-04 | 8 | 199 |
| antigen receptor-mediated signaling pathway | 2.04E-02 | 5 | 86 |
| mature B cell differentiation | 2.04E-02 | 3 | 12 |
| immune response-activating cell surface receptor signaling pathway | 2.76E-02 | 5 | 99 |
| positive regulation of immune response | 3.29E-02 | 7 | 292 |
| immune response-regulating cell surface receptor signaling pathway | 3.29E-02 | 5 | 111 |
| GTPase activator activity | 3.29E-02 | 5 | 113 |

Supplemental Table 4. Summary of real-time RT-PCR validation of selected differentially expressed genes that were identified by microarray analysis in four groups of mice. Fold changes were calculated based on average relative expression of each gene in 6 mice from each group. GAPDH was used as a reference gene for RT-PCR. All gene expression changes in RT-PCR are consistent with microarray analysis. * Indicates p<0.05; ** indicates p<0.01 based on unpaired *t* test.

| Groups | Mouse Gene Symbol | Gene Name | Fold Changes | | | |
| --- | --- | --- | --- | --- | --- | --- |
|  |  |  | CS vs. Air | | +/- vs. +/+ | |
|  |  |  | Hhip+/+ | Hhip+/- | Air | CS |
| Gene-by-treatment interaction genes | Adh7 | alcohol dehydrogenase 7 (class IV), mu or sigma polypeptide | 7.32****** | 1.14 | 2.24****** | 0.35****** |
|  | Arhgap4 | Rho GTPase activating protein 4 | 0.53****** | 1.45***** | 0.81 | 2.68****** |
|  | Azgp1 | alpha-2-glycoprotein 1, zinc-binding | 1.33 | 0.80 | 1.71****** | 1.03 |
|  | Fyb | FYN binding protein | 0.44 | 1.70****** | 0.41 | 1.61***** |
|  | Glipr2 | GLI pathogenesis-related 2 | 0.48****** | 1.20 | 0.53****** | 1.32***** |
|  | Hs3st1 | heparin sulfate (glucosamine) 3-O-sulfotransferase 1 | 1.57****** | 0.69 | 1.88****** | 0.82 |
|  | Itgb7 | integrin beta 7 | 0.74***** | 1.69****** | 0.95 | 2.17****** |
|  | Slc11a1 | solute carrier family 11 (proton-coupled divalent metal ion transporters), member 1 | 0.26***** | 1.65***** | 0.30 | 1.93****** |
|  | Tef | thyrotrophic embryonic factor | 1.87****** | 0.62 | 3.25** | 1.08 |
|  | Wisp2 | WNT1 inducible signaling pathway protein 2 | 0.70 | 1.45 | 1.30 | 2.68** |
|  | Lat2 | linker for activation of T cells family, member 2 | 0.73* | 1.71* | 0.88 | 2.05** |
|  | Gstp1 | Glutathione S-Transferase Pi 1 | 1.69* | 1**.41*** | 0.83 | 0.69** |
| Non gene-by-environment interaction genes | Mt3 | metallothionein 3 | 0.47 | 0.70 | 4.68** | 6.91* |
|  | Spon2 | spondin 2, extracellular matrix protein | 0.26** | 0.40** | 0.32** | 0.48** |
|  | Gap43 | growth associated protein 43 | 0.59** | 0.76 | 0.58** | 0.75 |
|  | Dbp | D site albumin promoter binding protein | 3.38* | 1.05 | 6.72** | 2.08 |
|  | Ctsw | Cathepsin W | 0.661* | 1.64* | 1.06 | 1.73** |
|  | Crem | cAMP responsive element modulator | 0.62* | 0.79 | 0.49** | 0.63** |

Supplemental Table 5. Characteristics of subnetworks in four pair-wise comparisons based on PANDA analysis:

| Network Comparison (Network 1 vs. Network 2) | Number of Edges in Subnetworks (Network 1/Network 2) | Number of Genes Targeted in Subnetworks (Network 1/Network 2) | Number of TFs Targeting in Subnetworks (Network 1/Network 2) |
| --- | --- | --- | --- |
| Hhip ^+/-^ Air vs. Hhip^+/+^ Air | 9008/10105 | 1353/2057 | 33/29 |
| Hhip ^+/-^ CS vs. Hhip^+/+^ CS | 12572/11952 | 2091/1843 | 32/30 |
| Hhip ^+/-^ CS vs. Hhip^+/-^ Air | 8982/8826 | 1642/1486 | 24/28 |
| Hhip ^+/+^ CS vs. Hhip^+/+^ Air | 13723/16918 | 2087/2954 | 39/33 |

Supplemental Table 6. Transcription factors (TFs) regulating differential subnetworks in *Hhip^+/-^*-Air and *Hhip^+/+^-*Air subnetworks identified by PANDA analysis.

|  | Edge Numbers | | | log2 Fold Change *Hhip^+/-^*-Air vs, *Hhip^+/+^-*Air | -log10 p value |
| --- | --- | --- | --- | --- | --- |
| TFs | *Hhip^+/-^*-Air | *Hhip^+/+^-*Air | Total |
| FOXJ1 | 12 | 0 | 12 | Infinite | 3.92 |
| NKX3-1 | 17 | 0 | 17 | Infinite | 5.56 |
| SOX7 | 4 | 0 | 4 | Infinite | 1.31 |
| FOXI1 | 45 | 3 | 48 | 4.07 | 11.29 |
| ELF5 | 25 | 2 | 27 | 3.81 | 6.15 |
| PAX4 | 34 | 3 | 37 | 3.67 | 8.03 |
| SOX11 | 37 | 5 | 42 | 3.05 | 7.50 |
| SOX4 | 72 | 11 | 83 | 2.88 | 13.35 |
| SRY | 26 | 4 | 30 | 2.87 | 5.11 |
| HNF4A | 88 | 16 | 104 | 2.63 | 14.75 |
| FOXD3 | 147 | 32 | 179 | 2.37 | 21.58 |
| MZF1_5-13 | 11 | 3 | 14 | 2.04 | 1.77 |
| EBF1 | 6 | 2 | 8 | 1.75 | 0.96 |
| ZFP740 | 256 | 106 | 362 | 1.44 | 19.39 |
| ZFP105 | 385 | 204 | 589 | 1.08 | 18.95 |
| TBP | 289 | 178 | 467 | 0.86 | 10.19 |
| FOXA2 | 296 | 220 | 516 | 0.59 | 5.83 |
| ZFP281 | 598 | 482 | 1080 | 0.48 | 7.84 |
| SOX5 | 277 | 230 | 507 | 0.43 | 3.45 |
| SP4 | 733 | 973 | 1706 | -0.24 | 3.78 |
| KLF7 | 808 | 1083 | 1891 | -0.26 | 4.54 |
| KLF4 | 1001 | 1343 | 2344 | -0.26 | 5.61 |
| SP1 | 1176 | 1667 | 2843 | -0.34 | 10.90 |
| ZFX | 343 | 509 | 852 | -0.40 | 4.67 |
| GABPA | 299 | 533 | 832 | -0.67 | 10.78 |
| ELK4 | 79 | 154 | 233 | -0.80 | 4.58 |
| ELK1 | 203 | 403 | 606 | -0.82 | 11.48 |
| TP53 | 1 | 3 | 4 | -1.42 | 0.45 |

Supplemental Table 7: Transcription factors (TFs) in *Hhip^+/-^-*CS and *Hhip^+/+^-*CS subnetworks identified by PANDA analysis.

| TFs | Edge numbers | | | log2 Fold Change *Hhip^+/-^*-CS vs. *Hhip^+/+^*-CS | -log10 p value |
| --- | --- | --- | --- | --- | --- |
|  | *Hhip^+/-^*-CS | *Hhip^+/+^*-CS | Total |  |  |
| ELF2 | 6 | 1 | 7 | 2.51 | 1.15 |
| ZFP161 | 32 | 10 | 42 | 1.61 | 3.09 |
| ZIF268 | 12 | 4 | 16 | 1.51 | 1.32 |
| SP1 | 1965 | 1630 | 3595 | 0.20 | 5.25 |
| EGR1 | 1613 | 1344 | 2957 | 0.19 | 4.13 |
| KLF4 | 1726 | 1479 | 3205 | 0.15 | 3.06 |
| ZFP740 | 312 | 385 | 697 | -0.38 | 3.55 |
| TBP | 52 | 87 | 139 | -0.82 | 3.17 |
| ARID3A | 298 | 565 | 863 | -1.00 | 23.30 |
| SOX5 | 63 | 120 | 183 | -1.00 | 5.53 |
| FOXA2 | 62 | 166 | 228 | -1.49 | 13.01 |
| ZFP105 | 92 | 260 | 352 | -1.57 | 21.46 |
| FOXD3 | 8 | 35 | 43 | -2.20 | 4.99 |
| SOX4 | 1 | 7 | 8 | -2.88 | 1.52 |
| ELF5 | 0 | 5 | 5 | -Infinite | 1.56 |

Supplemental Table 8 Transcription factors (TFs) regulating differential subnetworks in *Hhip^+/-^*-CS and *Hhip^+/-^*-Air subnetworks identified by PANDA analysis.

| TFs | Edge Numbers | | | log2 Fold Change *Hhip^+/-^-*CS vs. *Hhip^+/-^-*Air | -log10 p value |
| --- | --- | --- | --- | --- | --- |
|  | *Hhip^+/-^-*CS | *Hhip^+/-^-*Air | Total |  |  |
| ELK4 | 252 | 96 | 348 | 1.37 | 16.55 |
| ELK1 | 421 | 194 | 615 | 1.09 | 19.59 |
| GABPA | 547 | 259 | 806 | 1.05 | 24.02 |
| ZFX | 614 | 435 | 1049 | 0.47 | 7.44 |
| KLF7 | 1089 | 877 | 1966 | 0.29 | 5.75 |
| SP1 | 1511 | 1264 | 2775 | 0.23 | 5.64 |
| KLF4 | 1274 | 1087 | 2361 | 0.20 | 3.89 |
| EGR1 | 1158 | 999 | 2157 | 0.19 | 3.16 |
| ZFP281 | 492 | 618 | 1110 | -0.35 | 4.84 |
| ZFP740 | 120 | 216 | 336 | -0.87 | 7.54 |
| ARID3A | 204 | 652 | 856 | -1.70 | 59.25 |
| FOXA2 | 74 | 263 | 337 | -1.85 | 26.73 |
| SOX17 | 63 | 234 | 297 | -1.92 | 24.90 |
| HNF4A | 3 | 12 | 15 | -2.03 | 1.79 |
| SOX5 | 59 | 242 | 301 | -2.06 | 28.17 |
| ZFP105 | 58 | 247 | 305 | -2.12 | 29.68 |
| TBP | 25 | 155 | 180 | -2.66 | 24.39 |
| MZF1_5-13 | 2 | 16 | 18 | -3.03 | 3.24 |
| FOXD3 | 5 | 63 | 68 | -3.68 | 13.68 |
| SOX4 | 1 | 14 | 15 | -3.83 | 3.36 |
| EBF1 | 0 | 4 | 4 | - Infinite | 1.22 |
| FOXI1 | 0 | 6 | 6 | -Infinite | 1.83 |
| PAX4 | 0 | 9 | 9 | -Infinite | 2.74 |
| SOX11 | 0 | 7 | 7 | - Infinite | 2.13 |

Supplemental Table 9 Transcription factors (TFs) in *Hhip^+/+^-*CS and *Hhip^+/+^-*Air identified by PANDA analysis.

| TFs | | Edge Number | | | | | | log2 Fold Change *Hhip^+/+^-*CS vs. *Hhip^+/+^-*Air | | -log10 p value | |
| --- | --- | --- | --- | --- | --- | --- | --- | --- | --- | --- | --- |
|  |  | *Hhip^+/+^-*CS | | *Hhip^+/+^-*Air | | Total | |  |  |  |  |
| ASCL2 | | 6 | | 0 | | 6 | | Infinite | | 2.09 | |
| FOXJ1 | | 6 | | 0 | | 6 | | Infinite | | 2.09 | |
| PAX4 | | 15 | | 0 | | 15 | | Infinite | | 5.23 | |
| PPARG::RXRA | | 15 | | 0 | | 15 | | Infinite | | 5.23 | |
| SOX11 | | 14 | | 0 | | 14 | | Infinite | | 4.89 | |
| SOX4 | | 53 | | 4 | | 57 | | 4.03 | | 13.92 | |
| FOXI1 | | 23 | | 2 | | 25 | | 3.83 | | 6.04 | |
| ELF5 | | 61 | | 10 | | 71 | | 2.91 | | 12.16 | |
| FOXD3 | | 131 | | 36 | | 167 | | 2.17 | | 18.29 | |
| MZF1_5-13 | | 36 | | 14 | | 50 | | 1.66 | | 4.05 | |
| HNF4A | | 145 | | 63 | | 208 | | 1.50 | | 12.55 | |
| EBF1 | | 41 | | 19 | | 60 | | 1.41 | | 3.71 | |
| ZFP105 | | 415 | | 235 | | 650 | | 1.12 | | 22.36 | |
| FOXA2 | | 237 | | 166 | | 403 | | 0.82 | | 8.05 | |
| ZFP740 | | 388 | | 354 | | 742 | | 0.43 | | 4.71 | |
| ZFX | | 770 | | 1100 | | 1870 | | -0.21 | | 3.20 | |
| ELK1 | | 462 | | 746 | | 1208 | | -0.39 | | 5.80 | |
| ELK4 | | 233 | | 389 | | 622 | | -0.44 | | 3.96 | |
| SOX17 | | 153 | | 316 | | 469 | | -0.74 | | 7.38 | |
| TP53 | | 2 | | 16 | | 18 | | -2.70 | | 2.58 | |
| NR1H2::RXRA | | 1 | | 18 | | 19 | | -3.87 | | 3.69 | |
| ZFP161 | 0 | | 4 | | 4 | | - Infinite | | 1.03 | |  |
